# Proximity biotinylation at the host-*Shigella* interface reveals UFMylation as an antibacterial pathway

**DOI:** 10.1101/2025.05.29.656827

**Published:** 2025-05-29

**Authors:** Ana T. López-Jiménez, Fabien Théry, Kathryn Wright, Hannah Painter, Shelby T. Hoffmeister, Lucas Jarche, Jeremy Benjamin, Gerbrand J. van der Heden van Noort, Dominik Brokatzky, Margarida C. Gomes, Sydney L. Miles, Damián Lobato-Márquez, John Rohde, Jonathan N. Pruneda, Francis Impens, Serge Mostowy

**Affiliations:** 1: London School of Hygiene and Tropical Medicine, Keppel St, London WC1E 7HT, United Kingdom.; 2: VIB-UGent Center for Medical Biotechnology, VIB, Technologiepark-Zwijnaarde 75, 9052 Ghent, Belgium.; 3: UGent Department of Biomolecular Medicine, Ghent University, Technologiepark-Zwijnaarde, 9052 Ghent, Belgium.; 4: Department of Molecular Microbiology and Immunology, Oregon Health & Science University, 3181 SW Sam Jackson Park Road, Portland, Oregon 97239, United States of America.; 5: Department of Microbiology and Immunology, Dalhousie University. Sir Charles Tupper Medical Building, 5850 College Street, B3H 4R2 Halifax, Nova Scotia, Canada.; 6: Department of Cell and Chemical Biology, Leiden University Medical Centre, Einthovenweg 20, 2333 ZC Leiden, The Netherlands.; 7: Current address: Dynamics of host-pathogen interaction unit, Institut Pasteur, Université Paris Cité, 75015 Paris, France.; 8: Current address: School of Life Sciences, University of Warwick, CV4 7AL, Coventry, UK.; 9: Current address: Centro Nacional de Biotecnología, Consejo Superior de Investigaciones Científicas (CNB-CSIC), C/ Darwin 3, 28049 Madrid, Spain.

**Keywords:** bacterial infection, cell-autonomous immunity, E3 ligases, proximity biotinylation, *Shigella*, ubiquitin-like systems, UFMylation

## Abstract

Host cells contest invasion by intracellular bacterial pathogens with multiple strategies that recognise and / or damage the bacterial surface. To identify novel host defence factors targeted to intracellular bacteria, we developed a versatile proximity biotinylation approach coupled to quantitative mass spectrometry that maps the host-bacterial interface during infection. Using this method, we discovered that intracellular *Shigella* and *Salmonella* become targeted by UFM1-protein ligase 1 (UFL1), an E3 ligase that catalyses the covalent attachment of Ubiquitin-fold modifier 1 (UFM1) to target substrates in a process called UFMylation. We show that *Shigella* antagonises UFMylation in a dual manner: first, using its lipopolysaccharide (LPS) to shield from UFL1 recruitment; second, preventing UFM1 decoration by the bacterial effector IpaH9.8. Absence of UFMylation leads to an increase of bacterial burden in both human cells and zebrafish larvae, suggesting that UFMylation is a highly conserved antibacterial pathway. Contrary to canonical ubiquitylation, the protective role of UFMylation is independent of autophagy. Altogether, our proximity mapping of the host-bacterial interface identifies UFMylation as an ancient antibacterial pathway and holds great promise to reveal other cell-autonomous immunity mechanisms.

*Shigella flexneri* is a human-adapted enteroinvasive pathogen that causes over 200 million episodes of bacillary dysentery annually worldwide^1^. Its virulence relies on a Type 3 Secretion System (T3SS) that injects around 30 bacterial effectors into host cells to modulate key aspects of the infection process^2,3^. Once in the gastrointestinal tract, *S. flexneri* invades colonic epithelial cells and escapes from the phagosome to the cytosol within 10 minutes^4^. *S. flexneri* has evolved to thrive in the host cytosol, where it replicates and hijacks the actin cytoskeleton to form distinctive actin tails that enable intracellular motility and cell to cell spread^5,6^.

Cells protect their nutrient-rich cytosol from bacterial intruders with a plethora of highly conserved mechanisms collectively known as cell-autonomous immunity^7^. These include the prevention of actin tail motility by the entrapment of *S. flexneri* in septin cage-like structures^8,9^ and guanylate-binding protein (GBP) coats^10,11^. GBPs have also been shown to bind and disrupt the bacterial lipopolysaccharide (LPS)^12^, which exposes bacterial membranes to permeabilisation by Apolipoprotein L3^13^. In addition, bacterial LPS becomes ubiquitylated by the E3 ubiquitin ligase ring finger protein 213 (RNF213), which is required for the subsequent extension of the ubiquitin bacterial coat by other host E3 ligases (LRSAM1, Parkin, LUBAC and SMURF1)^14^. Although bacterial ubiquitylation by E3 ligases typically targets intracellular bacteria such as *Salmonella* to degradation by autophagy (xenophagy) via specific cargo receptors, *S. flexneri* avoids autophagosome recruitment by the effectors IcsB, VirA, and likely other mechanisms yet to be understood^15–20^.

In addition to canonical ubiquitylation, eukaryotic cells possess multiple ubiquitin-like (UBL) systems. These are post-translational modification machineries where small peptides with low sequence homology but high structural similarity to ubiquitin (including the β-grasp fold or ubiquitin fold) become conjugated to target substrates by the sequential action of an E1 activating, an E2 conjugating and an E3 ligating enzyme^21^, in a similar process to ubiquitylation. Although several UBL systems (FAT10ylation^22^, ISGylation^23^, NEDDylation^24,25^, SUMOylation^26,27^) are protective against various bacterial infections, none of them have been shown to have a direct antibacterial role at the bacterial surface analogous to ubiquitylation.

Here, we develop a versatile nanobody-based proximity biotinylation approach to map host proteins enriched in the vicinity of cytosolic *S. flexneri* during infection using quantitative mass spectrometry. Using this method, we discover that *S. flexneri* and *Salmonella enterica* subsp. Enterica srv. Typhimurium become targeted by UFMylation, a highly conserved UBL system^28^ that has recently emerged as a major regulator of cell homeostasis. This is particularly well studied at the endoplasmic reticulum (ER), where UFMylation is involved in stress tolerance through ribosome-associated quality control^29^ and selective ER autophagy (ER-phagy)^30^. In addition, UFMylation has been shown to be involved in DNA damage responses^31^, telomere length maintenance^32^, lipid droplet biogenesis^33^, autophagy regulation^34^, and immune signalling^35,36^. Here, we show that UFMylation also has a cell-autonomous immunity role against intracellular bacteria. We show that *S. flexneri* counteracts UFMylation via two distinct mechanisms: its LPS hampers the recruitment of the E3 ligase UFL1, and the secreted effector IpaH9.8 antagonises decoration of the UBL molecule UFM1 on the surface of intracellular bacteria. While UFMylated bacteria are not targeted to degradation by autophagy, lack of UFMylation leads to increased bacterial burden in human epithelial cells and zebrafish larvae. Together, we identify UFMylation as a highly conserved cell-autonomous immunity pathway against bacterial infection.

## Results

### Proximity biotinylation approach at the host-bacterial interface identifies UFL1 as targeted to intracellular bacteria.

**Figure 1.**
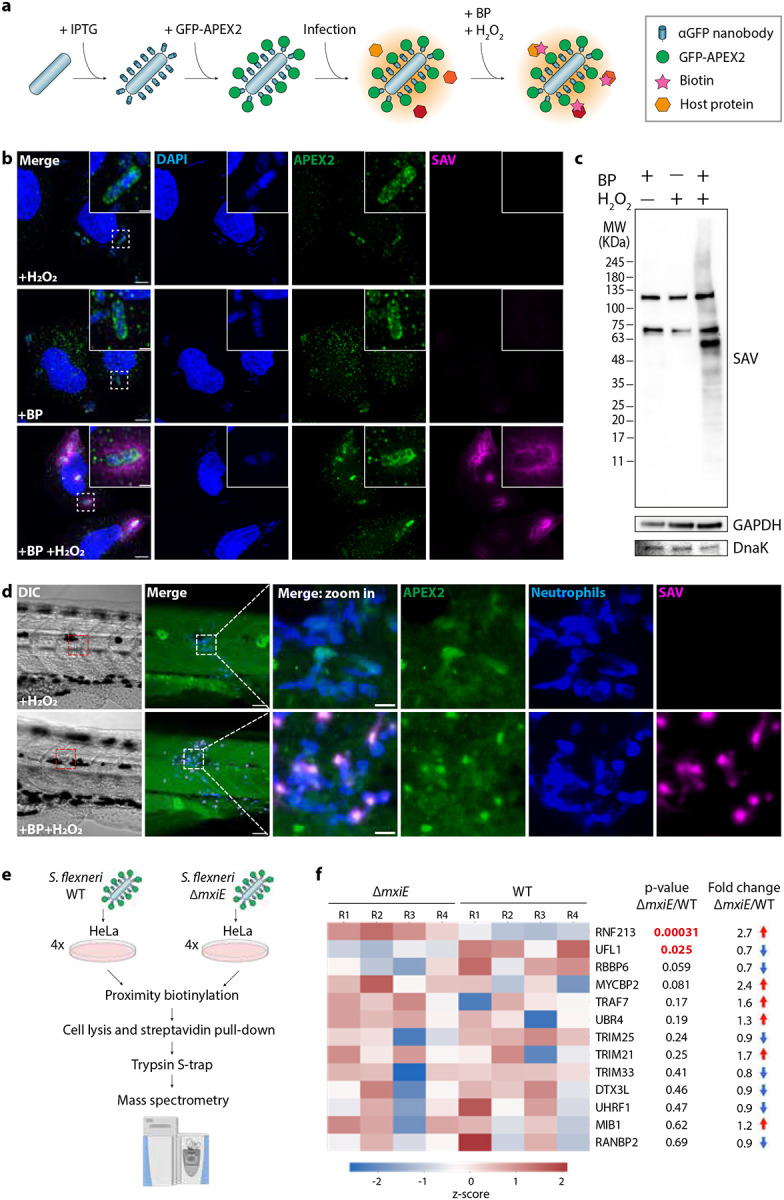
Novel proximity biotinylation approach to identify the *S. flexneri* proxisome during infection. **a,** Strategy for the in vitro functionalisation of *S. flexneri* for proximity mapping. First, an anti-GFP nanobody is displayed on the *S. flexneri* surface by induction with IPTG. After that, *S. flexneri* is coated with purified GFP-APEX2 in vitro. Then, functionalised bacteria are used for infection assays. Finally, the addition of biotin phenol (BP) and H_2_O_2_ enables the proximity biotinylation reaction during infection. **b,** Representative airyscan confocal images showing biotinylation in the vicinity of functionalised *S. flexneri* during infection in HeLa cells, in the presence or absence of BP and H_2_O_2_. SAV stands for streptavidin. Scale bar, 5 μm and 1 μm for the inset. **c,** Western blot shows a smear of biotinylated proteins in HeLa cells infected with functionalised *S. flexneri* upon addition of BP and H_2_O_2_. **d,** Proximity biotinylation occurs in zebrafish larvae at 45 minutes post infection of functionalised *S. flexneri* at the tail musculature. The transgenic zebrafish line *Tg(lyzC:DsRed2)*^*nz50*^ was used to visualise fluorescent neutrophils. Scale bar, 50 μm and 10 μm for the inset. **e,** Experimental workflow to identify the proxisome of *S. flexneri* WT and Δ*mxiE* mutant using mass spectrometry. Image created using BioRender. **f,** Heatmap of E3 ligases found in the proxisomes of *S. flexneri* during infection, inversely ordered by p-value.

Recognition and restriction of intracellular pathogens by cell-autonomous immunity provides an immediate and localised defence against infection before the organism can mount an immune response. Importantly, many cell-autonomous immunity mechanisms that host cells deploy against bacterial intruders are delivered to the bacterial surface. We exploited this feature to develop a proximity biotinylation approach that specifically maps host proteins at the interface between the host and the bacterial surface during *S. flexneri* infection (i.e., the bacterial “proxisome”). For this, we engineered *S. flexneri* to display anti-GFP nanobodies on its surface, which enabled their functionalisation in vitro with GFP-fused recombinant proteins such as APEX2, an engineered ascorbate peroxidase widely used for proximity biotinylation campaigns^37^ (Fig. 1a and Extended Data Fig. 1a, b). In a second step, bacteria functionalised with GFP-APEX2 can be used in infection assays for the biotinylation of host proteins in close proximity to bacteria and subsequent identification by mass spectrometry. In vitro, we observed specific biotinylation of the *S. flexneri* surface when the anti-GFP nanobody was expressed and exogenous biotin-phenol and H_2_O_2_ were added (Extended Data Fig. 1c–e). We showed that GFP-APEX2 functionalised bacteria were able to infect HeLa cells and form actin tails and septin cages, which are considered hallmarks of the *S. flexneri* cytosolic lifestyle (Extended Data Fig. 2a, b). In HeLa, biotinylated proteins appeared as a halo around the bacteria during infection in immunofluorescence (Fig. 1b) and as a smear during western blot (Fig. 1c). Biotinylation of the *S. flexneri* microenvironment also occurred in vivo during infection of zebrafish larvae, highlighting the versatility of the method beyond tissue culture cells (Fig. 1d and Supplementary Video 1).

Using this approach, we identified the proxisome of *S. flexneri* during infection in HeLa cells by label-free quantitative mass spectrometry. We compared the proxisomes generated by wildtype (WT) bacteria and bacteria lacking the transcriptional regulator MxiE, which controls expression of late T3SS effectors including important factors that antagonise cell-autonomous immunity (Fig. 1e and Extended Data Fig. 2c, d). To this end, HeLa cells were infected with GFP-APEX2 functionalised WT or Δ*mxiE S. flexneri* and biotinylated proteins were pulled-down for liquid chromatography-tandem mass spectrometry (LC-MS/MS analysis, Fig. 1e). We identified and quantified a total of 1.298 proteins of which 9 were *S. flexneri* proteins and 1.289 were human host proteins (Supplementary Table 1 and Extended Data Fig. 3). GO term enrichment analysis of the host proteins identified multiple pathways related to host-pathogen interactions relevant for *S. flexneri* infection, including actin filament-based transport, outer-mitochondrial membrane organisation or stress granule assembly (Extended Data Fig. 3c). In addition, proteomic screening revealed that multiple members of the ubiquitylation machinery are recruited to the bacterial surface during infection, including 13 E3 ligases (Fig. 1f, and Extended Data Fig. 3d). One of the most enriched hits in the proxisome of the *S. flexneri* Δ*mxiE* was the E3 ligase ring finger protein 213 (RNF213), which was discovered to ubiquitylate the LPS of cytosolic *S*. Typhimurium^14^ and *S. flexneri*^18–20^. It has been recently shown that the MxiE-dependent effector IpaH1.4 prevents RNF213 recruitment to *S. flexneri*, explaining why RNF213 is enriched to Δ*mxiE* compared to WT bacteria in our proximity data. Considering the pivotal role of ubiquitin E3 ligases in host defence, we decided to focus on the E3 UFM1-protein Ligase 1 (UFL1, Fig. 1f), an enzyme that catalyses the covalent attachment of Ubiquitin-like modifier 1 (UFM1) to target proteins in a process called UFMylation similar to ubiquitylation. UFL1 had the second lowest p-value among the E3 ligases identified (Fig. 1f). Together, we show that our proximity biotinylation approach is effective to identify novel host factors in close proximity to *S. flexneri* during infection, including UFL1 and other E3 ligases.

### *S. flexneri* LPS shields intracellular bacteria from recognition by the UFMylation ligase UFL1.

**Figure 2.**
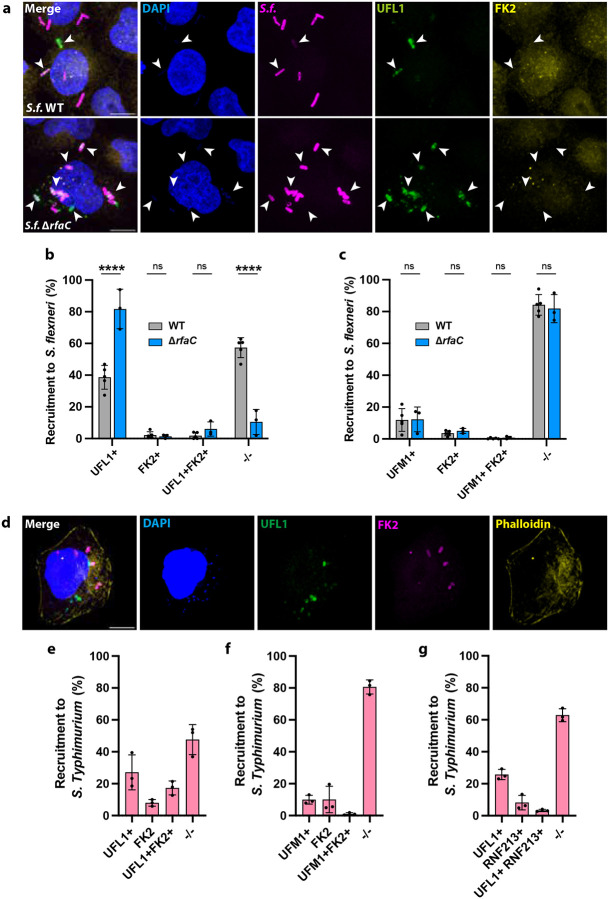
Several members of the UFMylation pathway are recruited to *S. flexneri* and *S*. Typhimurium during infection. **a,** Representative confocal images of HeLa cells infected with *S. flexneri* WT and Δ*rfaC*. Arrows show UFL1 recruitment to intracellular bacteria. Scale bar, 10 μm. **b,** Percentage of *S. flexneri* WT (n=1,311) and Δ*rfaC* (n=630) colocalising with UFL1 and ubiquitin (FK2), from at least 3 biological replicates. Two-way ANOVA and Tukey’s multiple comparison test. **c,** Percentage of *S. flexneri* WT (n=882) and Δ*rfaC* (n=643) colocalising with UFM1 and ubiquitin (FK2), from at least 3 biological replicates. Two-way ANOVA and Tukey’s multiple comparison test. **d,** Representative confocal image of HeLa cell infected with *S*. Typhimurium and stained for UFL1 and ubiquitin (FK2). Scale bar, 10 μm. **e,** Percentage of *S*. Typhimurium (n=740) colocalising with UFL1 and ubiquitin (FK2) **f,** Percentage of *S*. Typhimurium (n=823) colocalising with UFM1 and ubiquitin (FK2). **g,** Percentage of *S*. Typhimurium (n=735) colocalising with UFL1 and GFP-RNF213. The results are represented as mean ± SD.

Microscopy experiments validated the localisation of UFL1 and UFM1 to the surface of *S. flexneri* during infection in HeLa cells, using both HA-tagged exogenous expression and antibodies against the endogenous proteins (Fig. 2a–c and Extended Data Fig. 4). Considering that LPS is the bacterial substrate for ubiquitylation by the E3 ligase RNF213^14^, we tested the impact of UFL1 and UFM1 recruitment to a *S. flexneri* mutant Δ*rfaC*, which lacks the O-antigen and the LPS outer core^38^. We observed a strong increase in UFL1 recruitment in *S. flexneri* Δ*rfaC* (2.1 ± 0.2-fold, Fig. 2a, b and Extended Data Fig. 5d), suggesting that LPS protects intracellular bacteria from UFL1 binding. However, this increase in UFL1 binding did not correlate with an increase in UFM1 decoration of *S. flexneri* (WT 12.2% ± 5.0%, Δ*rfaC* 13.1% ± 8.5% bacteria, Fig. 2c and Extended Data Fig. 5e), suggesting that additional bacterial factors may prevent bacterial UFMylation. In both *S. flexneri* WT and Δ*rfaC* we observed similar and low values of ubiquitylation (WT: 3.9% ± 2.9 and 6.6 ± 3.6 bacteria, respectively), and there was little overlap between UFM1 and ubiquitin positive bacteria (Figure 2c), consistent with IpaH1.4 promoting RNF213 degradation^18–20^.

We hypothesised that the UFMylation machinery may recognise other intracellular bacteria beyond *S. flexneri*. We tested *S*. Typhimurium, a bacterium that typically resides within vacuoles but occasionally escapes into the cytosol, where it hyperreplicates^39^. We observed that UFL1 and UFM1 were also recruited to the surface of *S*. Typhimurium at 3 hours post infection (Fig. 2d–g), indicating that bacterial UFMylation is not a phenotype exclusive to *S. flexneri*. Most bacteria recruiting UFL1 or UFM1 did so in the absence of GFP-RNF213 recruitment or ubiquitylation (Fig. 2d–g and Extended Data Fig. 5f). Despite some overlap between UFL1 and FK2 decoration (17.3% ± 4.4% of the bacteria), robust recruitment of UFL1 and FK2 rarely coincided (Fig. 2d). Together, these results suggest that UFMylation and ubiquitylation are functionally separate machineries.

### UFMylation of intracellular *S. flexneri* is antagonised by the bacterial effector IpaH9.8.

**Figure 3.**
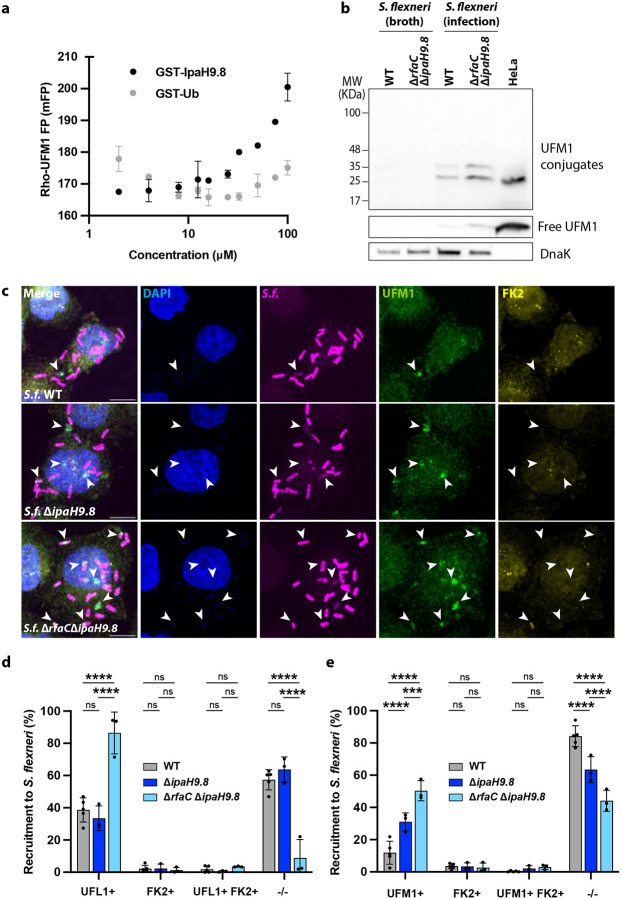
The *S. flexneri* secreted effector IpaH9.8 prevents UFM1 conjugation of intracellular bacteria. **a,** Protein-protein interaction assay monitoring fluorescence polarisation (FP) of rhodamine-labelled UFM1 in response to increasing concentrations of recombinant GST-IpaH9.8. To control for possible interactions with the GST tag of GST-IpaH9.8, GST-ubiquitin is titrated instead. **b,** Western blot showing UFM1 conjugates in *S. flexneri* WT and Δ*rfaC*Δ*ipaH9.8* purified from infection that are absent in bacteria from broth. UFM1 species present in uninfected HeLa cells are shown as a control. Different exposure times were used to detect free UFM1 (low) and UFM1 conjugates (high) due to relative differences in abundance, using anti-UFM1 antibodies (Abcam). **c,** Representative confocal images of HeLa cells infected with *S. flexneri* WT, Δ*ipaH9.8* and Δ*rfaC*Δ*ipaH9.8*. Arrows show UFL1 recruitment to intracellular bacteria. Scale bar, 10 μm. **d,** Percentage of *S. flexneri* WT (n=1,311), Δ*ipaH9.8* (n=463) and Δ*rfaC*Δ*ipaH9.8* (n=553) colocalising with UFL1 and ubiquitin (FK2). **e,** Percentage of *S. flexneri* WT (n=882), Δ*ipaH9.8* (n=476) and Δ*rfaC*Δ*ipaH9.8* (n=526) colocalising with UFM1 and ubiquitin (FK2). Two-way ANOVA and Tukey’s multiple comparison test. UFL1 and UFM1 quantifications for WT *S. flexneri* are the same as presented in Fig. 2b, c. The results are represented as mean ± SD.

The limited UFM1 decoration of *S. flexneri* during infection led us to hypothesise that additional bacterial effectors may intersect with the UFMylation cascade beyond UFL1 recruitment. Among T3SS effectors, members of the Invasion Plasmid Antigen H (IpaH) family of proteins have been shown to have important roles in counteracting cell-autonomous immunity mechanisms^40–42^. Importantly, a Yeast-Two-Hybrid screening for the *S. flexneri* secreted effector IpaH9.8 identified UFM1 as potential interaction partner (Supplementary Table 2), suggesting that IpaH9.8 may prevent *S. flexneri* UFMylation. Immunoprecipitation assays using recombinantly expressed IpaH9.8 enhanced the recovery of UFM1 species (Extended Data Fig. 6). Binding studies using purified proteins confirmed a weak but direct interaction between GST-tagged IpaH9.8 and UFM1 (Fig. 3a). Meanwhile, as a control, GST-tagged ubiquitin showed no interaction with UFM1. To understand the interaction of *S. flexneri* with UFM1, we isolated bacteria from infected cells and probed for the presence of UFM1 conjugates by western blot (Fig. 3b). We detected several UFM1 positive bands that were absent in bacteria from broth, indicating UFM1 conjugates at the bacterial surface. Interestingly, we observed a similar pattern in a *S. flexneri* double mutant Δ*rfaC*Δ*ipaH9.8* (Fig. 3b and Extended Data Fig. 5a, c), suggesting that LPS is not the bacterial substrate for UFMylation and that IpaH9.8 does not prevent the conjugation of specific substrates. To test a role for IpaH9.8 in counteracting *S. flexneri* UFMylation, we scored UFL1 and UFM1 recruitment to *S. flexneri* WT, Δ*ipaH9.8* and the double mutant Δ*rfaC*Δ*ipaH9.8*. While deletion of *ipaH9.8* did not affect UFL1 recruitment to *S. flexneri*, we observed a higher decoration of UFM1 in *S. flexneri* Δ*ipaH9.8* compared to WT at 3 hours post infection (2.6 ± 0.5-fold), which further increased (4.4 ± 0.6-fold) in the case of the double mutant Δ*rfaC*Δ*ipaH9.8* (Fig. 3c–e, and Extended Data Fig. 5a–e). At later timepoints (5 hours post infection) the levels of UFM1 decoration were higher and non-significantly different for *S. flexneri* WT, Δ*ipaH9.8* and the double mutant Δ*rfaC*Δ*ipaH9.8* (37.8% ± 30.6%, 54.6 ± 26.7%, 60.6% ± 12.1%, respectively, Extended Data Fig. 5a–e). Consistent with previous reports^10^, deletion of *ipaH9.8* in the absence of IFNγ did not impact *S. flexneri* ubiquitylation (Fig. 3d, e), further suggesting that UFMylation operates independently of the ubiquitin axis. Together, these results indicate that the *S. flexneri* T3SS effector IpaH9.8 interacts with UFM1 to delay UFMylation of the bacterial surface during infection.

### UFMylation has a conserved antibacterial role independent of autophagy.

Considering the ubiquitous expression of the UFMylation pathway in human cells (Human Cell Atlas) and its high conservation among eukaryotes^28^, we decided to test the localisation of UFL1 and UFM1 to *S. flexneri* in THP-1 macrophages and zebrafish larvae. In both cases, UFL1 and UFM1 localised to *S. flexneri* (Extended Data Fig. 7), suggesting a broad and conserved role during infection.

**Figure 4.**
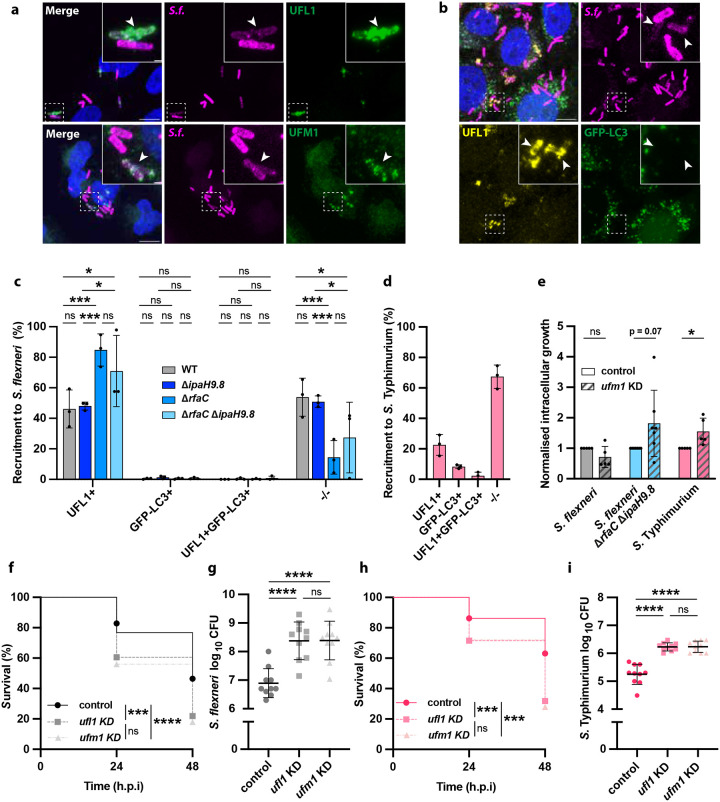
UFMylation has a conserved antibacterial role independent of autophagy. **a,** Representative confocal microscopy images of HeLa cells infected with *S. flexneri* mCherry and immunostained against UFL1 and UFM1. Arrows point at intracellular bacteria colocalising with UFL1 and UFM1 with a loss of mCherry fluorescence. Scale bar, 10 μm and 1 μm for the inset. **b,** Representative microscopy image of HeLa cells expressing GFP-LC3 infected with *S. flexneri* mCherry and immunostained against UFL1. Arrows indicate intracellular *S. flexneri* that recruit UFL1 and lose their mCherry fluorescence but are not engulfed in an autophagosome. Scale bar, 10 μm and 1 μm for the inset. **c,** Quantification of UFL1 and GFP-LC3 recruitment to *S. flexneri* WT (n=655), Δ*ipaH9.8* (n=733), Δ*rfaC* (n=422), and double Δ*rfaC*Δ*ipaH9.8* (n=627) mutants. Two-way ANOVA and Tukey’s multiple comparison test. **d,** Quantification of UFL1 and GFP-LC3 recruitment to *S*. Typhimurium (n=582). **e,** Intracellular growth of *S. flexneri* WT, Δ*rfaC*Δ*ipaH9.8* and *S*. Typhimurium in HeLa cells treated with *ufm1* siRNA normalised to control siRNA after 5 hours post infection. **f,** Zebrafish larvae survival after *S. flexneri* infection at the caudal vein for wildtype larvae (46.40%) and *ufl1* (21.85%) and *ufm1* (18.00%) KD crispants. Data was obtained from 76 wildtype, 81 *ufl1* and 70 *ufm1* KD crispant zebrafish larvae from two biological replicates. **g,** *S. flexneri* recovered from infected zebrafish larvae at 48 hours post infection (h.p.i) for wildtype larvae, *ufl1* and *ufm1* KD crispants. **h,** Zebrafish larvae survival after *S*. Typhimurium infection at the caudal vein for wildtype larvae (63.15%), *ufl1* (31.81%) and *ufm1* (27.91%) KD crispants. Data was obtained from 54 wildtype, 61 *ufl1* and 55 *ufm1* KD crispant zebrafish larvae from two biological replicates. **i,** *S*. Typhimurium recovered from infected zebrafish larvae at 48 hours post infection, for wildtype larvae, *ufl1* and *ufm1* KD crispants. The results are represented as mean ± SD.

We next tested whether UFMylation has an antibacterial role, similar to ubiquitylation. We observed that intracellular *S. flexneri* with a strong recruitment of UFL1 or UFM1 displayed a fluorescence loss of their mCherry constitutive reporter, suggesting UFMylated bacteria were subjected to degradation (Fig. 4a). Similar loss of fluorescence was also observed in THP-1 macrophages and zebrafish larvae (Extended Data Fig. 7). Considering ubiquitylation classically targets intracellular bacteria to degradation by the autophagic machinery, we assessed the colocalisation of UFMylation and the main autophagic marker GFP-LC3 during infection. In the case of *S. flexneri*, we observed no overlap between UFL1 recruitment and GFP-LC3 engulfment of intracellular bacteria (Fig. 4b, c), consistent with ubiquitylation results. *S. flexneri* mutants with increased UFL1 and/or UFM1 colocalisation (Δ*ipaH9.8*, Δ*rfaC*, Δ*rfaC*Δ*ipaH9.8*) did not increase the number of bacteria in autophagosomes (Fig. 4c). In the case of *S*. Typhimurium, most of the bacteria entrapped in an autophagosome also did not recruit UFL1 (Fig. 4d). Together, these results suggest that contrary to ubiquitylation, UFMylation of intracellular bacteria leads to bacterial degradation in a process independent of autophagy.

To further investigate the antibacterial role of the UFMylation pathway, we assessed its impact on *S. flexneri* and *S*. Typhimurium burden in cells (Fig. 4e and Extended Data Fig. 8). Depletion of *ufm1* using siRNA did not lead to an increase in intracellular bacterial replication in the case of *S. flexneri*, consistent with its capacity to antagonise the UFMylation pathway. *S. flexneri* Δ*rfaC*Δ*ipaH9.8* exhibited more pronounced growth upon *ufm1* depletion, but it was non-significant (p-value =0.070), suggesting that additional effectors may counteract this pathway. In the case of *S*. Typhimurium, a vacuolar pathogen with a limited arsenal for cytosolic survival, we observed a significant increase in intracellular replication upon *ufm1* knockdown (1.5 ± 0.4-fold, Fig. 4e and Extended Data Fig. 8). Together with the loss of fluorescence of UFMylation targeted cells, these results indicate that UFMylation plays a host defence role against cytosolic bacteria.

Finally, we decided to interrogate the role of the UFMylation pathway in vivo using the zebrafish infection model. We observed a strong decrease in zebrafish larvae survival during infection upon depletion of *ufm1* and *ufl1*, both in the case of *S. flexneri* and *S*. Typhimurium (Fig. 4f, h and Extended Data Fig. 9). This increased susceptibility correlated with increased bacterial load of more than an order of magnitude in both cases (Fig. 4g, i). Collectively, these results provide evidence for an important and conserved defence role against bacterial infection and highlight the UFMylation pathway as a conserved / ancient cell-autonomous immunity mechanism (Extended Data Fig. 10).

## Discussion

Proximity mapping techniques have emerged as key discovery tools in a wide variety of research fields. For infection biology, recent applications include screenings for novel host interacting partners of *Legionella*^43^ and *S*. Typhimurium^44^ secreted effectors, as well as the surface proteome (including host bound proteins) of *Vibrio cholerae* recovered from rabbit diarrheal fluid^45^. Here, we pioneered a proximity biotinylation approach to specifically map the host-bacterial interface during infection. This strategy is particularly relevant to understand the proteomic microenvironment of cytosol-dwelling bacteria, since the isolation and proteomic characterisation of bacterial containing vesicles (BCVs) has been performed successfully for multiple vacuolar pathogens^46–50^. In addition, our proximity biotinylation approach is highly versatile, as it is functional in tissue culture cells and in animal models, and it can be easily adaptable to any other genetically tractable Gram positive or negative bacteria.

Our proximity biotinylation screening identified multiple E3 ligases in close proximity to *S. flexneri* during infection. Among these, RNF213 was found enriched in bacteria lacking MxiE, an important transcriptional regulator for the expression of late T3SS effectors. Consistent with these results and serving as internal positive control for our screening, multiple studies have recently reported that *S. flexneri* deploys the bacterial effector IpaH1.4 (under MxiE transcriptional control) to target RNF213 to degradation^18–20^. The proxisome of *S. flexneri* also revealed UFL1, the only identified E3 ligase of the UFMylation pathway, the latest UBL system discovered.

Our work has shown that both UFL1 and its substrate peptide UFM1 are recruited to the surface of *S. flexneri* and *S*. Typhimurium during infection, which showed little to no overlap with bacterial ubiquitylation. The *S. flexneri* surface became UFMylated during infection, as bacteria recovered from infected cells presented UFM1-conjugates absent in bacteria from broth. In contrast to RNF213, which ubiquitylates LPS^14^, the nature of the UFMylated substrates on *S. flexneri* is likely proteinaceous, as they appeared as discrete, well-defined bands on western blot. This result indicates that bacterial ubiquitylation by RNF213 and UFMylation by UFL1 are separate pathways that protect the host cytosol in a multilayered manner.

*S. flexneri* has evolved strategies to avoid UFMylation, which underscores its relevance as an antibacterial pathway. First, the S*. flexneri* mutant Δ*rfaC* with shortened LPS showed an increased recruitment of UFL1, indicating that the LPS can shield intracellular bacteria from host recognition. It is worth noting that *S. flexneri* Δ*rfaC* is also more susceptible to septin cage entrapment^38^. Collectively, these results suggest that the protective role of the LPS may contribute not only to avoid extracellular recognition^51,52^, but also intracellular detection. Likely reflecting the host-pathogen arms race, host cells also evolved to recognise intracellular LPS by caspase-4/−11^53^ and the TRAF-interacting forkhead-associated protein A (TIFA)^54,55^ for inflammasome and NF-κB activation, respectively. In this context, it is not surprising that bacterial pathogens, including *S. flexneri*, tightly modulate their LPS production during infection^56,57^.

A second strategy deployed by *S. flexneri* to counteract UFMylation is the secreted effector IpaH9.8, which did not impact UFL1 recruitment to the bacteria but their decoration with UFM1. This adds to the list of known functions of IpaH9.8, which has been shown to dampen the host inflammatory response^58,59^ and interfere specifically with established cell-autonomous immune programs by targeting GBPs to proteasomal degradation^10,11^. It is remarkable how IpaH9.8 has evolved for such level of pleiotropy, and future structural and biochemical studies will delve into the specificity and molecular mechanism underlying the IpaH9.8 – UFM1 interaction.

Our work has revealed UFMylation as a novel cell-autonomous immune pathway against cytosolic bacteria. This pathway is active during infection of human cells (epithelial and THP-1 macrophages) and also in zebrafish larvae. Together with the high degree of conservation of the UFMylation cascade among eukaryotes^28^, this strongly suggests a broader antibacterial role across evolution. Remarkably, UFMylation has very recently been shown to participate in the regulation of interferon and NF-κB mediated responses during viral and bacterial infections^35,60–63^, which may support the different susceptibility to *S. flexneri* and *S*. Typhimurium observed in cellulo and in vivo. Taken together, the UFMylation machinery should now be recognised as a major contributor to host defence.

## Methods

### Reagents.

The following primary antibodies were used: mouse anti-Ubiquitinylated proteins, clone FK2 (Sigma, #04–263), rabbit anti-UFL1 (Proteintech, #26087–1-AP), rabbit anti-SEPT7 (IBL, #18991), mouse anti-HA-Tag (6E2) (Cell Signaling, #2367), rabbit anti-Myc-Tag (71D10) (Cell Signaling, #2278), mouse anti-GFP (Abcam, #ab1218) and rabbit anti-IpaH^64^. Rabbit anti-UFM1 (Proteintech, #15883–1-AP) was used for immunofluorescence, and rabbit anti-UFM1 (Proteintech, #15883–1-AP; BostonBiochem, #AF8237; Abcam #109305) were used for western blot as indicated in the figure legends. The following secondary antibodies were used: Alexa Fluor-488, −555 and −647 conjugated goat anti-mouse antibodies (Invitrogen #A-11001, #A-21424 and #A-21236), and Alexa Fluor-488, −555 and −647 conjugated goat anti-rabbit antibodies (Invitrogen, #A-11008, #A-21428, # A-21244). The following reagents were used: HRP-conjugated streptavidin (Invitrogen, #S911), Alexa Fluor-488 conjugated streptavidin (Invitrogen, #S32354), Alexa Fluor-647 conjugated phalloidin (Invitrogen, #A22287) Congo red (Sigma-Aldrich, #C6767) and IFNγ (R&D Systems, #285-IF).

### Bacterial strains and culture conditions.

The bacterial strains and plasmids described in this study are listed in Supplementary Table 3. *Shigella flexneri* 5a str. M90T was grown in trypticase soy broth (TCS) agar containing 0.01% of Congo red to select for red colonies, indicative of a functional T3SS. Conical polypropylene tubes containing 5 ml of TCS were inoculated with individual red colonies of *S. flexneri* and were grown ~16 h at 37°C with shaking at 200 rpm. The following day, bacterial cultures were diluted in fresh prewarmed TCS (1:50 or 1:100 v/v), and cultured until an optical density (OD, measured at 600 nm) of 0.6. For *Salmonella enterica* subs. *enterica* srv. Typhimurium str. SL1344, single bacterial colonies were grown in conical polypropylene tubes containing 5 mL of LB for ~16 h at 37°C in static conditions. The following day, bacteria were subcultured in fresh prewarmed LB (1:50 or 1:100 v/v) and grown for 3h.

### Mammalian cell culture.

HeLa (ATCC #CCL-2) and HEK293T (ATCC #CRL-3216) were cultured in Dulbecco’s modified Eagle Medium (DMEM, GIBCO) in the presence of 10% heat-inactivated fetal bovine serum (hi-FBS, Gibco, #10500064) under standard conditions (37°C and 5% CO_2_). GFP–LC3B-producing HeLa cells^65^ were grown as above.

THP-1 cells (male monocytes, ATCC #TIB-202) were grown RPMI-1640 medium supplemented with 10% hi-FBS at 37°C and 5% CO_2._ THP-1 cells were differentiated via phorbol 12-myristate 13-acetate (PMA, Sigma-Aldrich, #P8139) treatment^66^ into macrophages using 100 nM PMA in RPMI-1640 with 10% hi-FBS for 48 h followed by 24 h recovery phase in RPMI with 10% hi-FBS.

### Cloning.

Plasmids used or generated in this study are listed in Supplementary Table 4.

Primers used in this study were designed using Benchling (https://benchling.com) or NEBuilder Assembly Tool (https://nebuilder.neb.com/#!/) and are listed in Supplementary Table 5.

For GFP-APEX2 purification, the pTRC-GFP-APEX2 plasmid was generated by Gibson assembly using pTRC-APEX2^37^ and pLVX-msGFP–SEPT6^38^ as template.

For Yeast-Two-Hybrid experiments, DNA fragment encoding IpaH9.8 C337A was amplified by PCR from plasmid pJR006^40^ and cloned as an EcoRI-BamHI fragment into the vector pGKBT7 to create the Yeast-Two-Hybrid bait vector pJB179–10.

For immunoprecipitation experiments, DNA fragments encoding IpaH9.8 and UFM1 were amplified by PCR and cloned as EcoRI-BamHI fragments into the vectors pRK5-Myc and pCR3.1 respectively to create pRK5Myc-IpaH9.8 and pCR3.1His-UFM1.

*S. flexneri* mutants were engineered using λ-Red-mediated recombination^67^. In brief, kanamycin resistance-encoding DNA cassettes were amplified using pKD4 plasmid as template and primers containing 50 bp nucleotides homologous to the site of insertion. Resulting DNA fragments were electroporated in *S. flexneri* electrocompetent cells producing λ-Red recombinase and plated in TSA plates supplemented with 0.01% of Congo red and 50 μg/ml of kanamycin. All strains were verified by PCR as shown in Extended Data Fig. 7. *S*. Typhimurium was transformed with the pmCherry plasmid, containing an *mCherry* gene controlled under a *P*_*BAD*_ arabinose-inducible promoter. *mCherry* was PCR-amplified from a pRK2-mCherry plasmid^68^ and cloned between ZraI and SpeI restriction sites in a pFUS-P_BAD_ plasmid^69^.

### siRNA treatment.

HeLa cells were transfected using siRNA targeting UFM1 (Thermo Fisher scientific, #148645) or using negative control siRNA (Thermo Fisher scientific, #4390843). HeLa cells were seeded in a 6-well plate and 200 nM of siRNA was transfected per well using Oligofectamine (Invitrogen, #12252011) as described by the manufacturer. Cells were incubated for 48 hours to ensure sufficient knockdown of targeted proteins.

### DNA transfections.

Hela and HEK273T cells were transfected using jetPEI transfection reagent (Polyplus, #101000053) according to the manufacturer’s instructions. Cells were incubated for 24 hours before the experiment.

### Protein purification.

For the in vitro binding of APEX2 to anti-GFP nanobodies displayed on the surface of *S. flexneri*, a monomeric superfolder version of GFP was added between the His_6_ tag and the N-terminus of APEX2 in the pTRC-APEX2 plasmid. GFP-APEX2 was purified as described previously^37,70^. Briefly, *Escherichia coli* BL21-DE3 cells transformed with the pTRC-GFP-APEX2 plasmid were grown to an OD (600 nm) of 0.5 in LB supplemented with 10 μg/mL ampicillin at 37°C with shaking at 200 rpm. Protein expression was induced with 1 mM IPTG (Sigma-Aldrich, #I5502) and supplemented with 1 mM 5-aminolevulinic acid hydrochloride (Sigma-Aldrich, #A7793) overnight at room temperature to promote heme incorporation. Harvested cells were lysed using B-PER in the presence of 1 mM PMSF (Thermo Fisher scientific, #36978) and cOmplete^™^ Protease Inhibitor Cocktail (Roche, #4693116001). The clarified lysates were incubated with Ni-NTA agarose chromatography matrix (Qiagen, #30210) for 30 minutes and loaded on an Econo-Pac column (Bio-Rad, #7321010) using gravity flow at 4 °C. The matrix was washed with binding buffer (50 mM Tris-HCl, 300 mM NaCl, pH 7.8) and washing buffer (binding buffer with 30 mM imidazole). GFP-APEX2 was eluted in binding buffer containing 200 mM imidazole. Purified protein was dialysed in PBS at 4°C and concentrated using a 30 KDa Amicon Ultra centrifugal filter unit (Millipore, #Z648035).

Expression plasmids for GST-IpaH9.8 and GST-ubiquitin, both in the pGEX6P-1 vector, were kind gifts from Dr. Neal Alto and Dr. David Komander, respectively. Protein expression was performed as described above with the exception of using Rosetta *E. coli* induced with 0.2 mM IPTG overnight at 18 °C. Cells were resuspended in 25 mM Tris-HCl, 200 mM NaCl, 2 mM *β*-mercaptoethanol, pH 8.0 and lysed by sonication in the presence of lysozyme, DNase, and protease inhibitor cocktail. The clarified lysates were loaded onto GST agarose (Pierce) in an Econo-Pac column for 60 minutes at 4 °C. The matrix was washed with the lysis buffer and eluted in lysis buffer containing 50 mM glutathione. Purified protein was dialysed in 25 mM Tris-HCl, 200 mM NaCl, 2 mM *β*-mercaptoethanol, pH 8.0 at 4°C and concentrated using a 30 KDa Amicon Ultra centrifugal filter unit (Millipore, #Z648035).

### In vitro coating of bacteria with GFP-APEX2.

*S. flexneri* WT or D*mxiE* mutant carrying pNVgfp plasmid for nanobody display on the bacterial surface^71^ and pAC-*afaI* plasmid to promote hyper invasion of HeLa cells^72^ were grown overnight in the presence of the appropriate antibiotics. The following day, bacteria cultures were diluted 1:50 and grown until OD (600 nm) 0.6 in the presence of the appropriate antibiotics and 1 mM of IPTG. 1.5 mL of bacterial culture was washed twice in PBS and incubated in 75 μL of PBS containing 300 nM of purified GFP-APEX2 at room temperature on rotation for 1 hour. Bacteria were washed twice with PBS to remove unbound GFP-APEX2 and used for subsequent infection assays.

### Proximity biotinylation.

Proximity biotinylation was adapted from previously published protocols^73^. 6 μM hemin-Cl (Sigma-Aldrich, #H9039) was added to all samples for the duration of the experiment to increase functionality of purified GFP-APEX2. For the biotinylation reaction, 500 μM of biotin-phenol (Sigma-Aldrich, #SML2135) was added to the sample and incubated for 30 minutes. 1 mM of H_2_O_2_ (Sigma-Aldrich, #H1009) was added and samples were incubated at 37°C for 1 minute (in vitro assay, in cellulo assay), or for 5 minutes (for zebrafish larvae). The biotinylation reaction was stopped by washing the cells in ice chilled quencher buffer containing 10 mM sodium ascorbate (Sigma-Aldrich, #A92902), 5 mM trolox (Sigma-Aldrich, #238813) and 10 mM sodium azide (Thermo Fisher Scientific #190380050) three times. Cells were collected using a cell scraper for western blot analysis or purification of biotinylated proteins. For immunofluorescence, samples were first fixed with 4% paraformaldehyde (in PBS) at room temperature and proximity biotinylation was performed on fixed cells or zebrafish larvae to prevent diffusion of biotinylated proteins.

### Purification of biotinylated proteins.

Purification of biotinylated proteins was performed as described before^73^. After proximity biotinylation, collected cells were centrifuged for 10 minutes at 3,000 xg at 4°C and lysed in RIPA buffer (750 μL for 10^7^ cells) supplemented with cOmplete^™^ Protease Inhibitor Cocktail, 1 mM PMSF and quenchers (10 mM sodium azide, 10 mM sodium ascorbate and 5 mM trolox), and incubated on ice for 2 minutes. Lysates were clarified by centrifugation at 15,000 xg for 10 minutes at 4 °C. Pierce^™^ Streptavidin magnetic beads (Thermo Fisher Scientific, #88816) were added to whole cell lysates (60 μL for 10^7^ cells) and incubated on rotation 1 hour at room temperature. A magnetic rack was used to pellet the beads and collect the supernatant (flow-through). Beads were washed to remove nonspecific binders in ice cold conditions twice with RIPA buffer, once with 1 M KCl, once with 0.1 M Na_2_CO_3_, once with 2 M urea in 10 mM Tris-HCl, pH 8.0, and twice with RIPA buffer again. Beads were snap frozen and subsequently analysed by mass spectrometry or alternatively processed for western blot analysis. For western blot, biotinylated proteins were eluted from the beads by boiling 50 μL of beads in 50 μL Laemmli buffer (3x) supplemented with 2 mM biotin and 20 mM DTT for 10 minutes and finally recovered from the beads using a magnetic rack.

### Sample preparation for LC-MS/MS analysis.

4 × 10^7^ HeLa cells were treated with 1 ng/uL IFNγ for 16 hours and were infected with GFP-APEX2 coated S*. flexneri* WT or Δ*mxiE* at MOI 80 in quadruplicates. Proximity biotinylation was performed at 1 hour 30 minutes post infection and biotinylated proteins were purified. Streptavidin beads were resuspended with 23 μL of a buffer containing 5% SDS (Merck, #L4509) with 100 mM triethylammonium bicarbonate (TEAB) (Merck, #T7408) at pH 8.0. Proteins were reduced and alkylated with 13 mM tris(2-carboxyethyl)phosphine (TCEP) (Thermo Fisher Scientific, #20491) combined with 40 mM chloroacetamide (CAA) (Merck, #C0267) and incubated for 10 minutes at 95°C under agitation. Samples were acidified by adding 2.5 μL of 27% Phosphoric acid (final 2.5%, v/v) (Merck, #438081). Samples were mixed with 90% methanol (Thermo Fisher Scientific, #A456–212) and 100 mM TEAB and loaded onto S-TrapTM micro columns (≤100 μg) (ProtiFi, #C2-micro) according to manufacturer’s protocol. Proteins were digested with 2 μg trypsin (#V5111, Promega) in 20 μL of 50 mM TEAB at pH 8.0 for overnight at 37°C on the S-trap. Peptides were eluted by the sequential addition of three elution buffers containing: 50 mM TEAB (buffer 1), 0.2% formic acid (FA) (Thermo Fisher Scientific, #270480010) (buffer 2) and 50% acetonitrile (ACN) (Thermo Fisher Scientific, #A955–212) with 0.2% FA (buffer 3). Peptides were dried under reduced pressure and desalted a second time using OMIX C18 pipette (#A57003MB, Agilent Technologies). Purified peptides were completely dried and stored at −20°C until LC-MS/MS analysis. Peptides were re-dissolved in 33 μL loading solvent (0.1% TFA in H_2_O/ACN, 99.5:0.5, v/v)) of which 2 μg of the sample measured on Dropsense16 (Unchained Labs) was injected for LC-MS/MS analysis on an Ultimate 3000 Pro Flow nanoLC system in-line connected to a Q Exactive HF mass spectrometer (Thermo Fisher Scientific).

### LC-MS/MS analysis.

Trapping was performed at 20 μL/min for 2 minutes in loading solvent on a 5 mm trapping column (Thermo Fisher Scientific, Pepmap, 300 μm internal diameter (I.D.), 5 μm beads). Peptides were separated on a 250 mm Aurora Ultimate, 1.7 μm C18, 75 μm inner diameter (Ionopticks) kept at a constant temperature of 45 °C. Peptides were eluted by a non-linear gradient starting at 0.5% MS solvent B (0.1% FA in ACN) and MS solvent A (0.1% FA in water). Gradient reached 33% at 70 minutes, 55% at 85 minutes and 70% at 90 minutes of MS solvent B. The gradient was followed by a 5-minute wash at 70% MS solvent B and re-equilibration with MS solvent A for 15 minutes. The mass spectrometer was operated in data-dependent mode (DDA), automatically switching between MS and MS/MS acquisition with a top 12 method. Full-scan MS spectra ranging from 375–1500 m/z with a target value of 3E6, a maximum fill time of 50 ms and a resolution at of 60,000 (at 200 m/z). Target accumulation was set at 3E6 for 60 ms in MS1. The 12 most intense ions above a threshold value of 1.3E4 were isolated in the trap with an isolation window of 1.5 Da for maximum 60 ms to a target AGC value of 1E3. Only precursor ions with a charge state equal to 2–6 were selected. Peptide match was set on “preferred” and isotopes were excluded. Dynamic exclusion time was set to 12 s. Fragmentation were performed at a normalised collision energy of 30% (NCE) with HCD fragmentation. MS/MS spectra were acquired at fixed first mass 145 m/z at a resolution of 15,000 (at 200 m/z) in the Orbitrap analyser. MS1 spectrum data type was set to profile MS2 spectrum data type was set to centroid. The polydimethylcyclosiloxane background ion at 445.12003 Da was used for internal calibration (lock mass) and QCloud was used to control instrument longitudinal performance during the project^74^.

### Data analysis for LC-MS/MS.

LC-MS/MS runs of all samples were searched using the MaxQuant algorithm (version 2.2.0.0)^75^. Spectra were searched against the protein sequences in the Swiss-Prot database (www.uniprot.org), from human (UP000005640_9606) and *Shigella flexneri* (strain 301 / Serotype 2a) (UP000001006_623) (downloaded in May 2023). Additionally, the database included the protein sequence for GFP-APEX2. Mainly default settings were used. The false discovery rate was set at 1% on peptide (precursor) and protein level. Enzyme specificity was set as C-terminal to arginine and lysine, also allowing cleavage at proline bonds with a maximum of two missed cleavages. Variable modifications were set to oxidation of methionine residues, acetylation of protein Ntermini and Biotin of lysine residues. Matching between runs was turn OFF. Proteins were quantified with MaxLFQ intensities based on razor and unique peptides with a minimum of two ratio counts per protein. Further data analysis was performed with an in-house script in the R programming language by loading the proteinGroups table from MaxQuant. Briefly, proteins identified in the reverse database were removed. Protein expression matrices were prepared as follows: Only proteins with intensity in three replicates were retained for analysis. Missing intensity values were imputed by randomly sampling from a normal distribution centered around each sample’s noise level. Fold change and adjusted p-value at FDR = 0.05 were calculated with the package Limma in R^76^. GO-term enrichment analysis was performed using the Term Enrichment Service from AmiGO^77^ (https://amigo.geneontology.org/amigo) and plotted using R.

### Yeast-Two-Hybrid Screening.

The Yeast-Two-Hybrid bait vector pJB79–10 encoding IpaH9.8 C337A was transformed into the Matchmaker Gold strain of *Saccharomyces cerevisiae* (Takara). A high-complexity normalised human cDNA Mate and Plate library (Takara) was screened following the protocol provided by the Matchmaker Gold Yeast-Two-Hybrid System (Takara). Positive prey clones were identified by plasmid rescue and DNA sequencing according as previously described^79^.

### Myc Fusion Protein Co-Immunoprecipitation of UFM1.

10^7^ HEK293T cells were transfected with equal amounts of pRK5Myc-IpaH9.8 and pCR3.1His-UFM1, or pCR3.1His-UFM1 alone as a control. Cells were collected in PBS 24 hours post-transfection, and washed once in PBS at 4°C. Samples were resuspended in 500 μL – 1mL of lysis buffer (2% SDS, 10% glycerol, 62.5 mM Tris pH 6.8), lysed with a 21G needle or with sonication and clarified by centrifugation at 13,000 xg for 15 minutes at 4°C. Supernatants were incubated overnight with 4 volumes of cold IP Buffer (50mM Tris pH8.0, 150mM NaCl, 1% NP-40) and αMyc-Tag (71D10) antibody (Cell Signaling, #2278) at 1:1000 dilution. The next day, 1 mL of Protein A agarose beads (Sino Biological, #10600-P07E) pre-washed with IP buffer was added to the lysates and incubated for 1–3 hours. Beads were collected by centrifugation at 700 xg for 2 minutes and washed three times with 1 volume IP buffer for 10 minutes. To elute bound proteins, beads were resuspended in 2x Laemmli buffer containing 10% *β*-mercaptoethanol and boiled for 5 minutes. Supernatant was collected by centrifugation at 300 xg for 2 minutes and analysed via SDS-PAGE.

### Fluorescence polarisation binding assay.

The fluorescent substrate Rhodamine-UFM1 (Rho-UFM1) was chemically synthesised as described previously^80^. Rho-UFM1, GST-IpaH9.8, and GST-ubiquitin were diluted to ‘2x’ concentrations in 25 mM Tris-HCl, 150 mM NaCl, 2 mM *β*-mercaptoethanol, 0.1 mg/mL BSA, pH 7.4. To set up the binding assay, 10 μL of 200 nM Rho-UFM1 was mixed with buffer or with 2x GST-tagged protein. The mixture was left to equilibrate in the dark at room temperature for 15 minutes, after which time it was loaded into black Greiner low-volume 384-well plates. Fluorescence polarisation values were measured on a BMG Labtech CLARIOstar plate reader equipped with 482–16 nm and 530–40 optic filters. A target polarisation value of 180 was set for free Rho-UFM1.

### Infection of HeLa cells.

Hela cells were seeded in 6-well plates (Thermo Fisher Scientific) at a confluency of 1.5 × 10^5^ cells/well 2 days prior to infection. Cell cultures were infected with *S. flexneri* strains as described previously^8^. Briefly, HeLa cells were infected with *S. flexneri* by spin-inoculation at 110 xg for 10 minutes at a multiplicity of infection (MOI) of 100:1 (bacteria:cell). Then, plates were placed at 37°C and 5% CO_2_ for 30 minutes. Infected cultures were washed three times with PBS pH 7.4 and incubated with fresh DMEM containing 10% hi-FBS and 50 μg/mL gentamicin at 37°C and 5% CO_2_ for up to 5 h. Infections with *S. flexneri* mutants lacking *rfaC* (hyperinvasive) or expressing the adhesin AfaE were performed at MOI 10 in the absence of spin inoculation, unless indicated otherwise.

For *S*. Typhimurium infections, 50 μL of bacterial subculture was added to wells containing HeLa cells and incubated for 30 minutes at 37°C. Following three washes with PBS, infected cultures were incubated with fresh DMEM containing 10% FBS and 50 μg/mL gentamicin at 37°C and 5% CO_2_ for up to 5 h.

### Intracellular bacteria growth.

HeLa cells seeded in 6-well plates and infected with *S. flexneri* were lysed in 1 ml cold PBS containing 0.1% Triton X-100. Serial dilutions were plated in duplicate on TCS agar containing 0.01% of Congo red for *S. flexneri* and LB agar for *S*. Typhimurium. Plates were incubated for 24 hours at 37°C and colony forming units (CFUs) enumerated. CFUs counted at 4 and 5 hours post infection were normalised to 1 hour post infection to assess intracellular growth.

### Bacteria recovery from infected cells.

HeLa cells were seeded into 15 cm dishes at a confluency of 5.0 × 10^6^ and infected two days later with the hyperinvasive *S. flexneri afaI* or Δ*rfaC*Δ*ipaH9.8* at MOI 10. Extraction of bacteria from infected cells was performed as described before^14^. Cells were lysed in ice cold PBS containing 0.1% Triton X-100 at 3 hours post infection and centrifuged at 300 xg for 5 minutes at 4 °C. Supernatant containing bacteria was collected and centrifuged at 16,100 xg for 10 minutes at 4 °C. The bacterial pellet was washed once with PBS, followed by bacterial lysis in 75 μl BugBuster (Merck, #70584) including 2 mM iodoacetamide for 5 minutes at room temperature. Samples were boiled after the addition of 25 μl of Laemmli buffer 4x for 10 minutes and analysed by western blot. As control, 1 mL of bacteria in TSB was collected by centrifugation at OD (600 nm) 0.6, washed twice with PBS and lysed as described above.

### Infection of THP-1 macrophages.

THP-1 cells were differentiated in 8-well μ-Slides (ibidi) at a confluency of 1 × 10^5^ cells per well. Cells were infected with *S. flexneri* strains with a multiplicity of infection (MOI) of 50. Plates were incubated for 30 minutes under standard conditions before being incubated with fresh RPMI supplemented with 10% hi-FBS and 50 μg/mL gentamycin under standard conditions for 30 minutes, followed by changing to RPMI supplemented with 10% hi-FBS containing 5 μg/mL gentamycin.

### Immunofluorescence of human cells.

Bacteria, coverslips or microslides containing adherent infected or uninfected human cells were washed three times with PBS pH 7.4 and fixed 15 minutes in 4% paraformaldehyde (in PBS) at room temperature. Fixed cells were washed three times with PBS and subsequently permeabilised 5 minutes with 0.1% Triton X-100 (in PBS). Cells were then washed three times in PBS and incubated 1 hour with primary antibodies diluted in PBS supplemented with 0.1% Triton X-100 and 1% bovine serum albumin (BSA, Sigma-Aldrich, #A9418) for 1 hour at room temperature or overnight at 4 °C. Cells were then washed three times in PBS and incubated for 1 hour with secondary goat antibodies diluted in PBS supplemented with 0.1% Triton X-100 and 1% BSA, and Alexa-conjugated phalloidin where indicated. Stained bacteria cultures and coverslips were washed three times with PBS and mounted on glass slides with ProLong^™^ Gold Antifade Mountant with DAPI stain (Invitrogen, # P36941).

### Detection of biotinylated proteins for fluorescence microscopy in human cells.

Fixed HeLa cells infected with GFP-APEX2 coated *S. flexneri* were permeabilised 5 minutes with 0.1% Triton X-100 (in PBS). Endogenous biotin was blocked with 0.05% avidin (Sigma-Aldrich, #A2667) in PBS for 15 minutes followed by 3 washes in PBS, and then 0.05% biotin (Sigma-Aldrich, #B4501) in PBS for 15 minutes followed by 3 washes in PBS. Then, proximity biotinylation was performed as described above. Biotinylated proteins were detected using Alexa Fluor-488 conjugated streptavidin and samples were further processed for immunofluorescence with anti-GFP antibodies to increase the detection of GFP-APEX2.

### Ethics statements.

Animal experiments were performed according to the Animals (Scientific Procedures) Act 1986 and approved by the Home Office (PPL PP5900632).

### Zebrafish husbandry.

Adult zebrafish were housed in the Biological Services Facility at LSHTM. Wildtype AB embryos and transgenic *Tg(lyzC:DsRed2)*^*nz50*^ embryos (for the expression of DsRed2 in neutrophils)^81^ were obtained by natural spawning, and larvae maintained at 28.5°C in E3 media (5 mM NaCl, 0.17 mM KCl, 0.33 mM CaCl_2_, 0.33 mM MgSO_4_). For injections, larvae were anesthetised with tricaine (160 μg/mL, Sigma-Aldrich, #A5040).

### CRISPR Cas9-mediated knockdown in zebrafish larvae.

Embryos were injected at the one-to two-cell stage with 1 nL of CRISPR mixture containing 1 μg/ μL of gene-specific Guide (g) RNA and 500 μg/mL Cas9 (spCas9 V3, IDT, #1081058) (Supplementary Table 5). gRNA was synthesised as previously described^82^. Briefly, primers containing gene-specific guide sites and a T7 promotor were annealed to a standard scaffold primer and purified (QIAquick PCR Purification Kit, Qiagen, #28104). DNA templates were in vitro transcribed (HiScribe T7 High Yield RNA Synthesis Kit NEB, #E2040S) and purified (GeneJET RNA Cleanup and Concentration Micro Kit, ThermoFisher, #K0841).

### Gene expression analysis.

Groups of 10 embryos were lysed and homogenised using a 27-gauge needle in Buffer RLT and RNA extracted with the RNeasy Mini kit (Qiagen, #74104). RNA was reverse transcribed using the QuantiTect Reverse Transcription kit (Qiagen, #205311), and quantitative (q) PCR performed on a QuantStudio Real-Time PCR system with SYBR green master mix (Applied Biosystems, #10187094). Samples were run in duplicates, and expression normalised to *ef1a1l*. Data was analysed using the 2^−ΔΔ^ Ct method. qPCR primers used are listed in Supplementary Table 5.

### Zebrafish injections.

To prepare the bacterial inocula, subcultures of either *S. flexneri* or *S*. Typhimurium at OD (600 nm) 0.6 were centrifuged at 4,000 x*g* for 4 minutes at room temperature and washed in PBS. The bacterial pellet was then resuspended in the appropriate volume of injection buffer (4% polyvinyl-pyrrolidone [Sigma-Aldrich, #PVP40] in PBS and 0.5% phenol red [Sigma-Aldrich, #114537] to obtain an infection dose of 10,000 CFU/nL for *S. flexneri* or 1,500 CFU for *S*. Typhimurium. For injection, 1 nL of bacterial suspension was microinjected into either the tail musculature (for immunofluorescence experiments) or caudal vein (for survival assays) of 3 days post fertilisation larvae.

### Wholemount zebrafish immunostaining.

Zebrafish larvae were fixed at 2 hours post infection in 4% paraformaldehyde with 0.4% triton X-100 and incubated at 4°C overnight. Larvae were then washed three times for five minutes in 0.4% Triton X-100 in PBS, followed by one 20-minute wash in 1% Triton X-100 in PBS. Samples were then blocked in blocking buffer (10% hi-FBS, 1% DMSO, 0.1% Tween-20 in PBS) for one hour at room temperature. Primary antibodies were prepared in blocking buffer and larvae were incubated overnight at 4°C with gentle rocking. Larvae were washed four times for 15 minutes with 0.1% Tween-20 in PBS, before incubation overnight at 4°C with secondary antibodies prepared in blocking buffer with gentle rocking. Larvae were washed as described and then cleared in a glycerol series of 25, 50 and 70%, allowing the larvae to sink to the bottom of the tube between each glycerol concentration. Larvae were mounted on 35-mm glass-bottom dishes (#P35G-1.5–14-C; MatTek) in a minimal volume of 70% glycerol for subsequent imaging.

### Detection of biotinylated proteins for fluorescence microscopy in zebrafish larvae.

Fixed zebrafish larvae infected with GFP-APEX2 coated *S. flexneri* were permeabilised with 0.4% triton X-100 and incubated at 4°C overnight. Larvae were then washed three times for five minutes in 0.4% Triton X-100 in PBS, followed by one 20-minute wash in 1% Triton X-100 in PBS. Endogenous biotin was blocked with 0.05% avidin in blocking buffer (10% hi-FBS, 1% DMSO, 0.1% Tween-20 in PBS) for one hour at room temperature, washed four times for 15 minutes, incubated with 0.05% biotin in blocking buffer for 1 hour at room temperature and washed four times for 15 minutes. Then, proximity biotinylation was performed as described above. Biotinylated proteins were detected using Alexa Fluor-488 conjugated streptavidin and samples were further processed for immunofluorescence with anti-GFP antibodies to increase the detection of GFP-APEX2.

### Bacterial recovery from zebrafish larvae.

At the required timepoint, 5 larvae per group were collected for CFU recovery. Larvae were euthanised by anaesthetic overdose (tricaine) and individually homogenised with a 27-gauge needle in 150 μL of PBS. Homogenates were serially diluted and plated on TSA plates supplemented with 0.01% Congo red, or LB agar plates. Plates were incubated for 24 hours at 37°C and CFU enumerated.

### Flow cytometry.

5 × 10^4^ individual bacterial cells were analysed using flow cytometry with an LSRII flow cytometer (BD Biosciences). The data was analysed using FlowJo software, version 10.7.1. The bacterial fluorescence was normalised to the median fluorescence of the control condition (no addition of GFP-APEX2) for each biological replicate. The normalised values, median and interquartile range was plotted.

### Secretion assay.

Secretion of T3SS effectors was performed as previously described^83^. Briefly, bacteria were grown overnight, subcultured and grown until OD (600nm) of 0.4–0.5 at 37°C. Cultures were then incubated for 3 hours in the presence or absence of Congo red to induce Type 3 secretion. Secreted proteins were collected from culture supernatants, precipitated using trichloroacetic acid (Sigma-Aldrich, #T0699), and then analysed using SDS-PAGE and Coomassie Brilliant Blue R-250 (Bio-Rad, #1610436) staining.

### SDS-PAGE.

Cells were lysed in ice-cold RIPA buffer and then incubated 1:1 with Laemmli buffer (2x) containing 10% (v/v) *β*-mercaptoethanol at 95°C for 10 minutes. Proteins were resolved in 12% or 4–20% Mini-PROTEAN TGX precast protein gels (Biorad, #4561046EDU, #4561096EDU).

### Western blotting.

Protein gels were transferred to 0.2 μm polyvinylidene difluoride membranes (PVDF, Biorad, #1704156). PVDF membranes were incubated in blocking buffer (TBST buffer containing 3% of BSA) for 1 hour. PVDF membranes were incubated with the primary antibodies diluted in blocking buffer for 1 hour at room temperature or overnight at 4 °C. PVDF membranes were washed 3× 5–7 minutes in PBS at room temperature and incubated with secondary goat HRP-conjugated antibodies in blocking buffer for 1 hour at room temperature. Milk was replaced by 3% BSA in the blocking buffer for the detection of biotinylated proteins with Streptavidin-HRP or detection of UFM1 with antibodies. Membranes were developed using Pierce^™^ ECL plus western blotting substrate (Thermo Scientific, #32132)

### Microscopy.

Fluorescence microscopy was performed using a 63×/1.4 C-Plan Apo oil immersion lens or a 40x/1.2 C-Apo water immersion lens on a Z-eiss LSM 880 confocal microscope driven by ZEN Black software (v2.3). Microscopy images were obtained using z-stack image series taking 8–16 slices (HeLa cells) or 40–120 slices (zebrafish larvae). When using the airyscan acquisition mode, confocal images were processed using airyscan processing (Weiner filter) using “Auto Filter” and “3D Processing” options. Alternatively, and for the purpose of quantification of host factors to bacteria, fluorescence microscopy of infected HeLa cells was performed on an AxioObserver Z1 fluorescence microscope driven by ZEN Blue v2.3 software (Carl Zeiss). Whole larvae images were acquired using stereo fluorescent microscope Leica M205FA (Leica, Germany). Image files were processed using ImageJ/Fiji software for representation.

### Statistics.

All graphs were plotted, and statistical analysis were performed using Prism. n.s: non-significant, *: p-value < 0.05, **: p-value < 0.01, ***: p-value < 0.001.

## Supplementary Material

Supplement 1

1
