# Supplementary figures and images for: Proximity biotinylation at the host-Shigella interface reveals UFMylation as an antibacterial pathway
Source: bioRxiv. 2025 May 29:2025.05.29.656827. Preprint. [Version 1] doi: 10.1101/2025.05.29.656827 (PMC12154702; doi:10.1101/2025.05.29.656827)

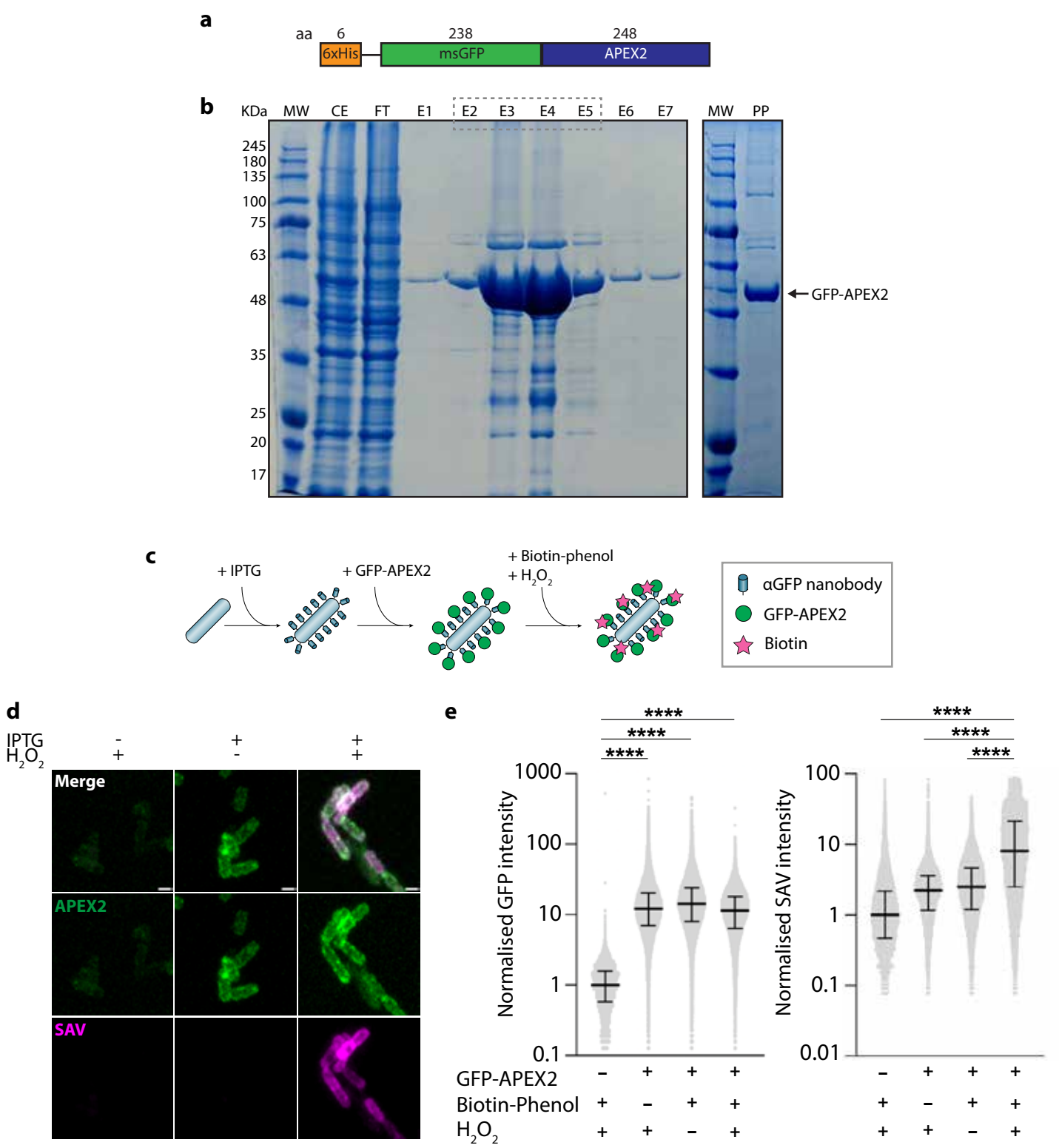

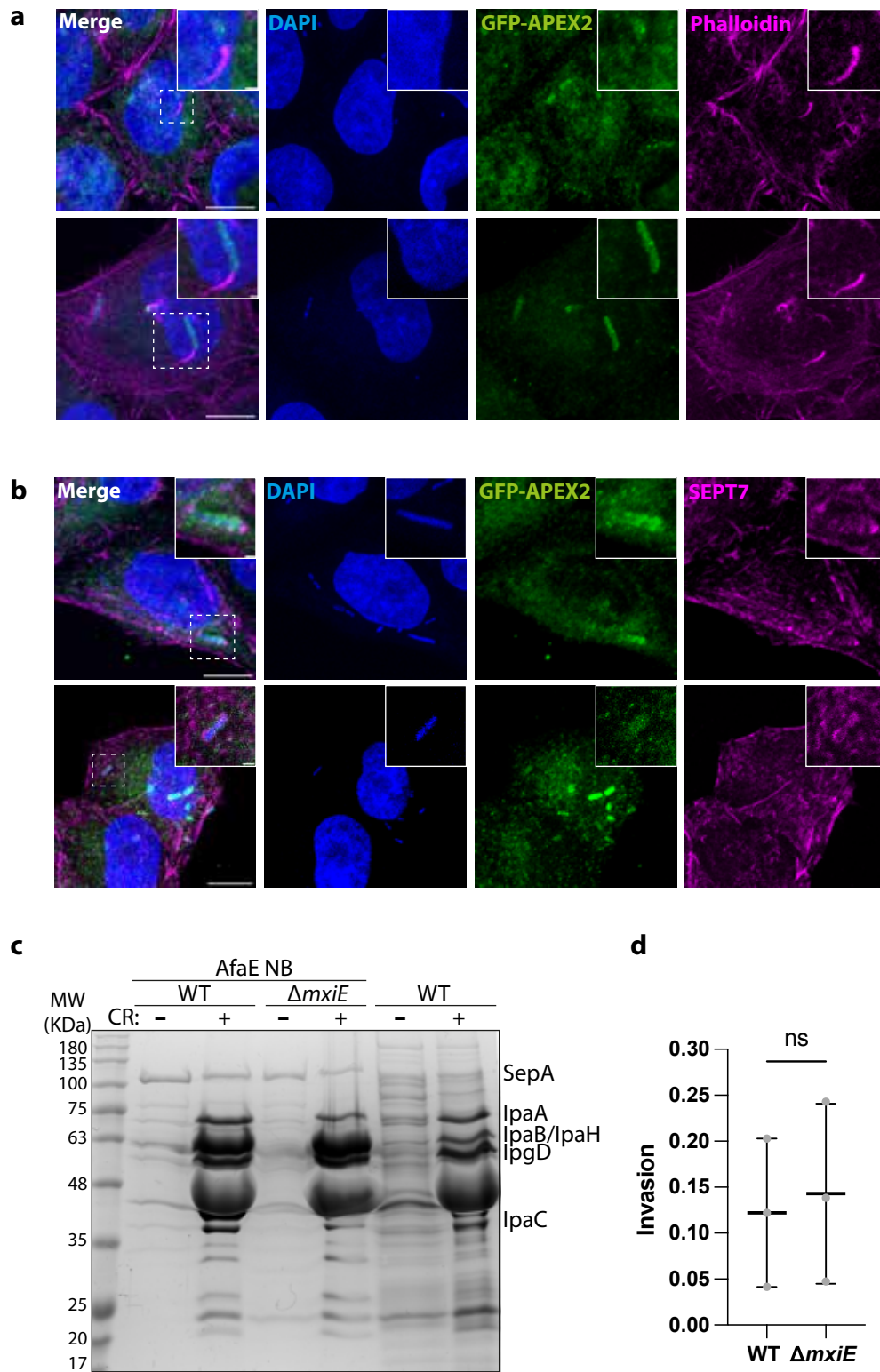

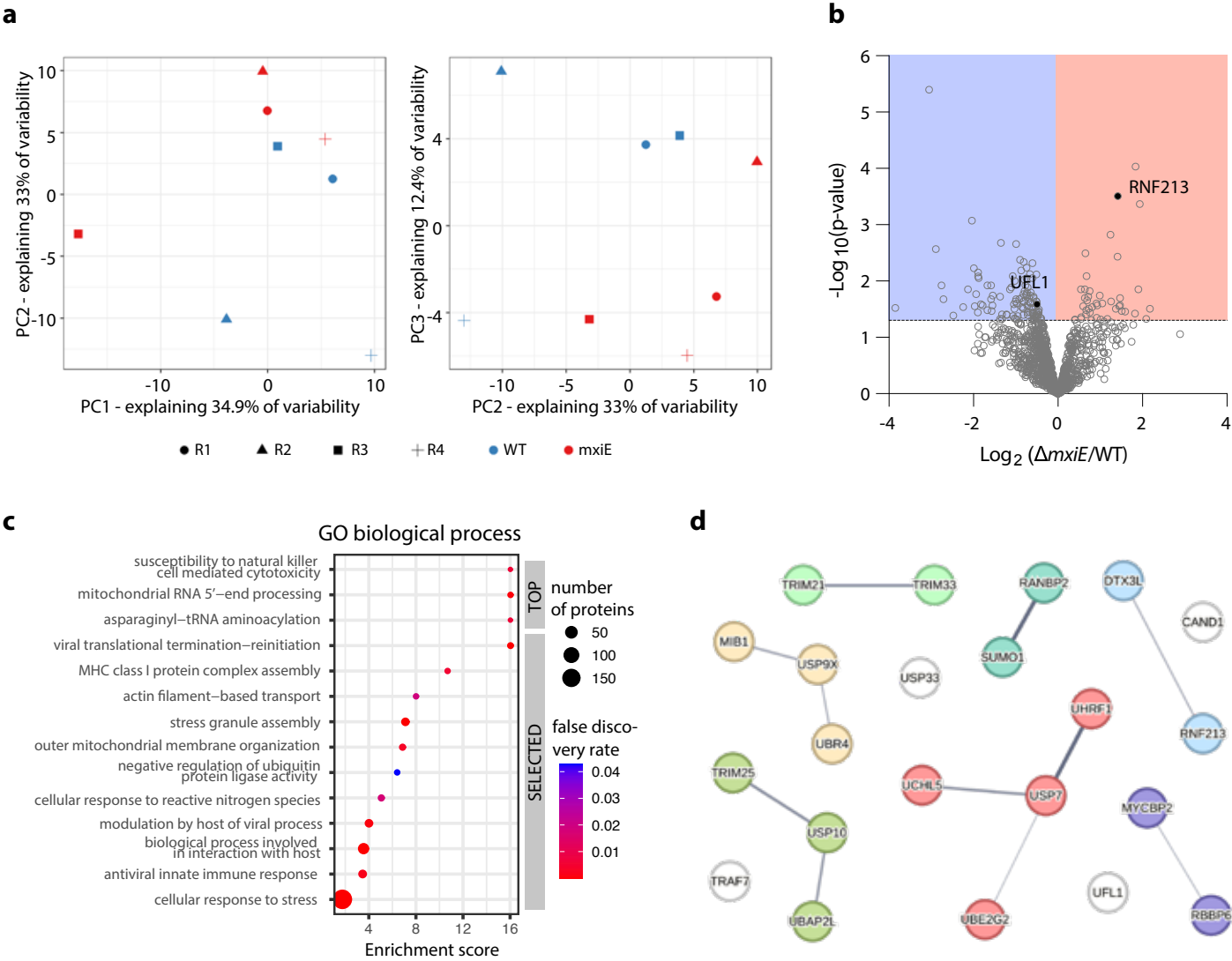

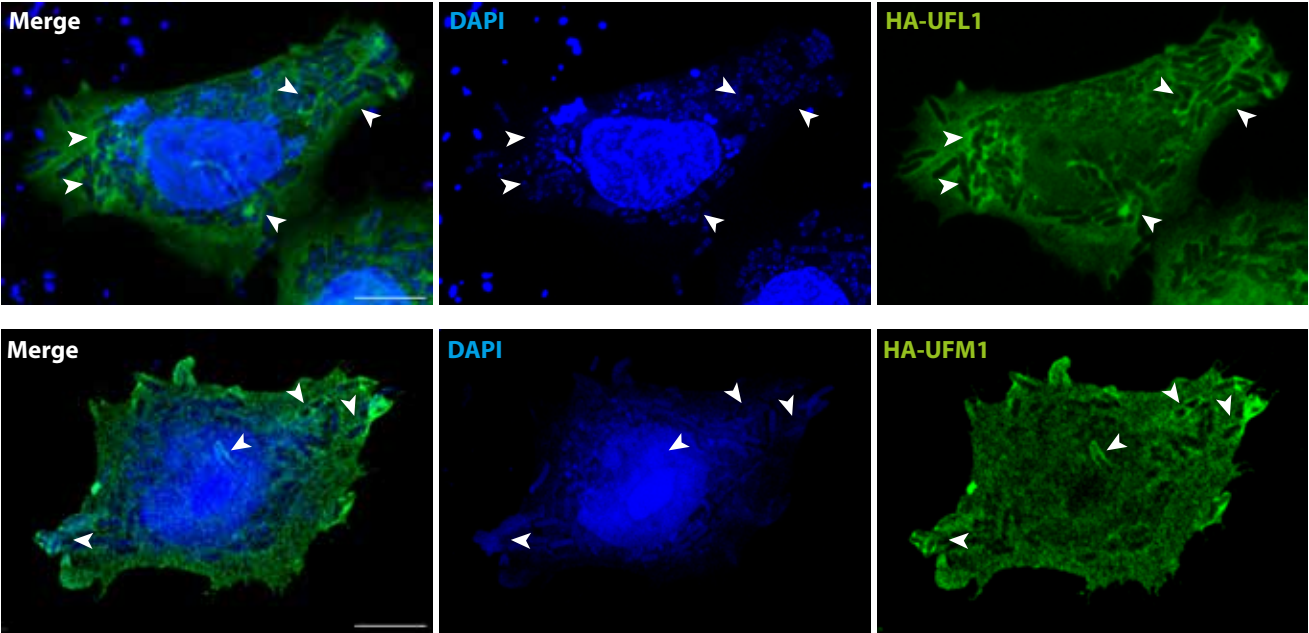

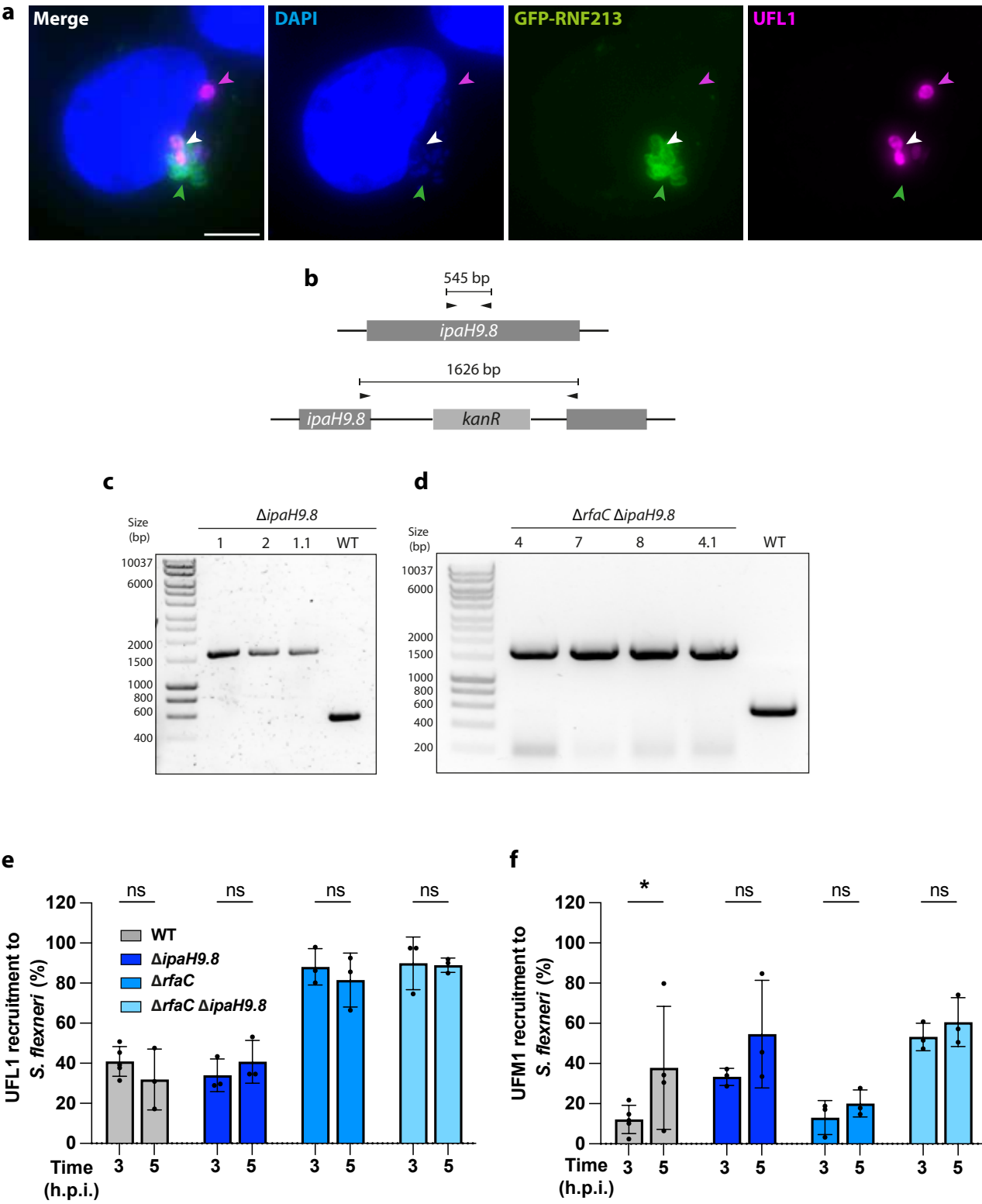

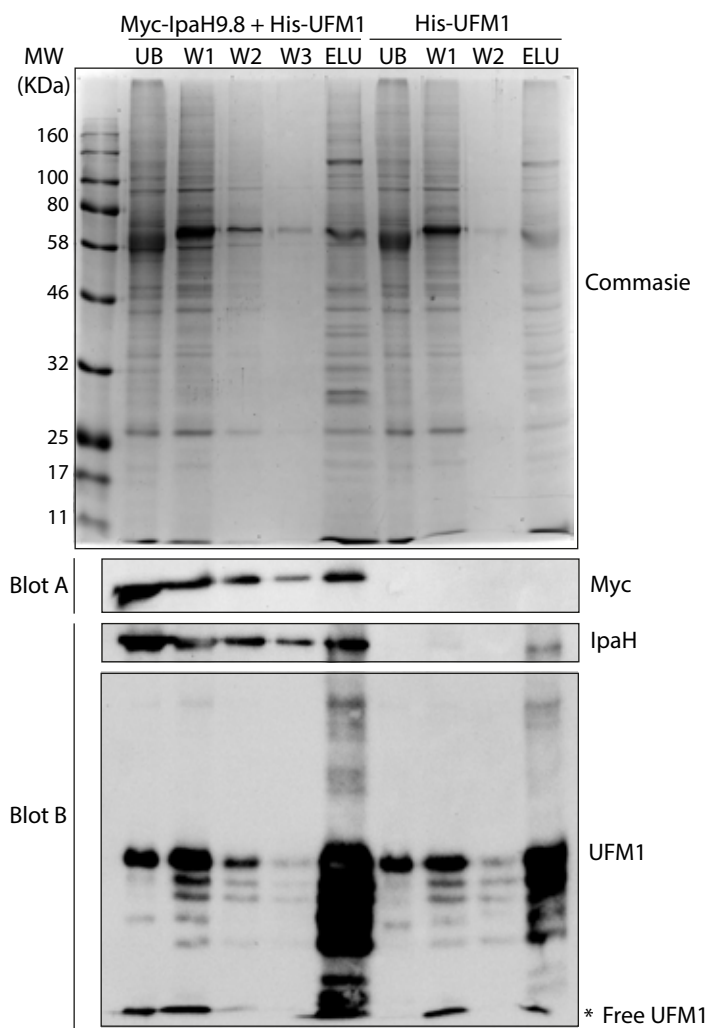

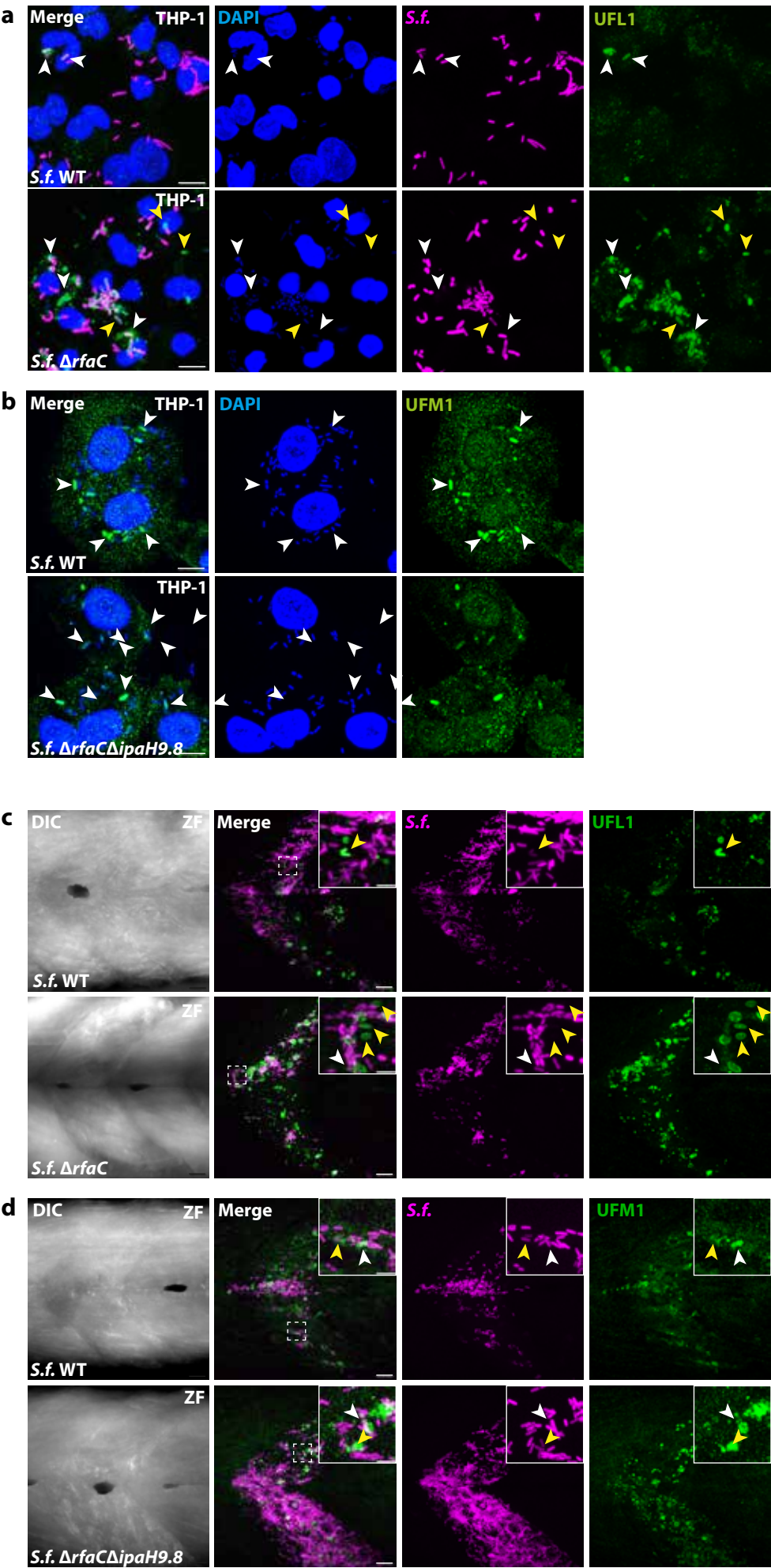

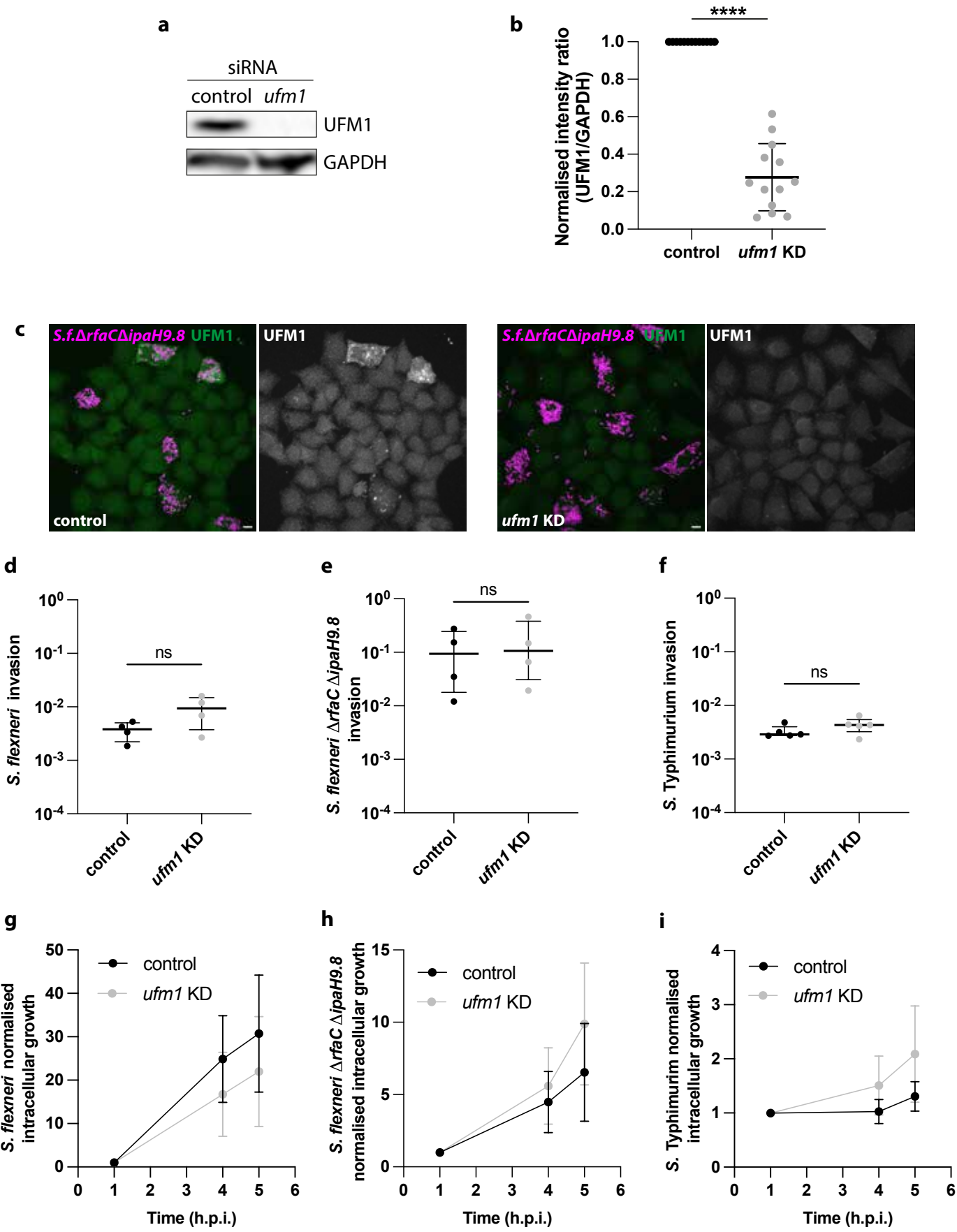

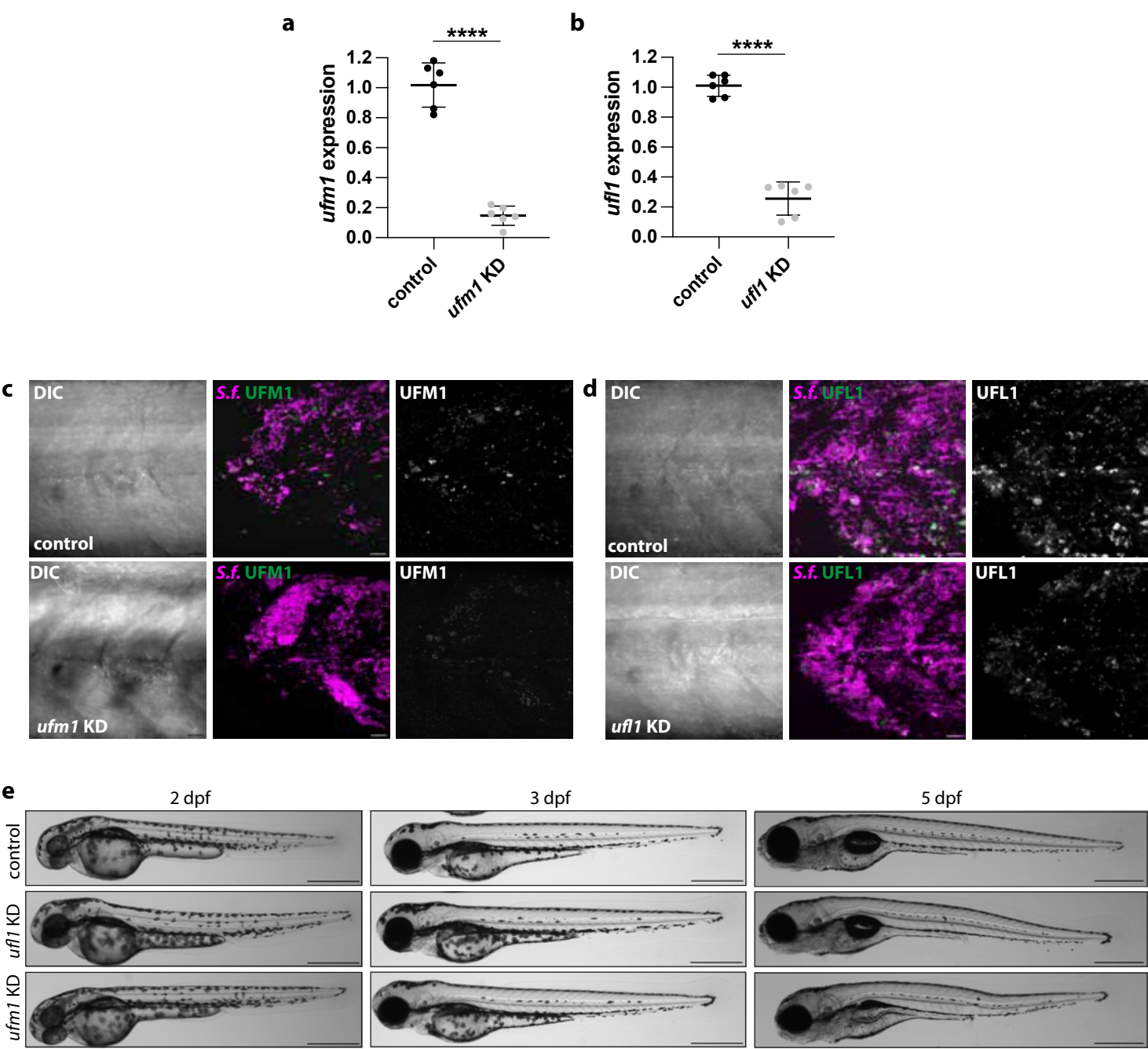

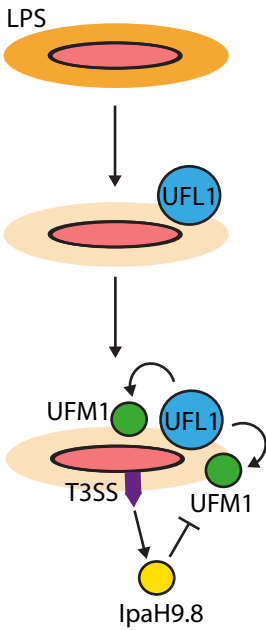

Supplement: Supplement 1 [file media-1.pdf]
