# Supplementary material for: Proximity biotinylation at the host-Shigella interface reveals UFMylation as an antibacterial pathway
Source: bioRxiv. 2025 May 29:2025.05.29.656827. Preprint. [Version 1] doi: 10.1101/2025.05.29.656827 (PMC12154702; doi:10.1101/2025.05.29.656827)
Supplement: 1 [file NIHPP2025.05.29.656827V1-supplement-1.pdf]

bioRxiv preprint doi: https://doi.org/10.1101/2025.05.29.656827; this version posted May 29, 2025. The copyright holder for this preprint (which was not certified by peer review) is the author/funder, who has granted bioRxiv a license to display the preprint in perpetuity. It is made available under aCC-BY 4.0 International license.

Tables

Supplementary Table 1. Mass spectrometry protein identification and quantification of *S. flexneri* WT or Δ*mixE* proxisomes during infection of HeLa cells. Proteins were identified with MaxQuant at 1% FDR to both peptides and protein level.

bioRxiv preprint doi: https://doi.org/10.1101/2025.05.29.656827; this version posted May 29, 2025. The copyright holder for this preprint (which was not certified by peer review) is the author/funder, who has granted bioRxiv a license to display the preprint in perpetuity. It is made available under aCC-BY 4.0 International license.

Supplementary Table 2. Top 50 mammalian proteins shown to interact with IpaH9.8 by Yeast-Two-Hybrid protein- protein interaction screen, ordered by the number of clones detected.

Frequency	Gene ID	Description
23	ATP1B3	ATPase, Na+/K+ transporting, beta 3 polypeptide
11	ARHGDIB	Rho GDP dissociation inhibitor (GDI) beta
7	KRT222	keratin 222
7	CCT3	chaperonin containing TCP1, subunit 3 (gamma)
6	ATP1B1	ATPase, Na+/K+ transporting, beta 1 polypeptide
6	MRVI1	murine retrovirus integration site 1 homolog
5	PCNA	proliferating cell nuclear antigen
5	ZKSCAN3	zinc finger with KRAB and SCAN domains 3
4	C17orf46	chromosome 17 open reading frame 46
4	PGM1	phosphoglucomutase 1
3	C9orf78	chromosome 9 open reading frame 78
3	GBP4	guanylate binding protein 4
3	EIF1B	eukaryotic translation initiation factor 1B
3	MAP3K1	mitogen-activated protein kinase kinase kinase 1
2	TMED7	transmembrane emp24 protein transport domain containing 7
2	MPHOSPH8	M-phase phosphoprotein 8
2	MYBPC1	myosin binding protein C, slow type
2	RPS20	ribosomal protein S20
2	ZBTB38	zinc finger and BTB domain containing 38
1	LOC284412	hypothetical LOC284412, non-coding RNA
1	PRDX6	peroxiredoxin 6
1	AP1G1	adaptor-related protein complex 1
1	C14orf45	gamma 1 subunit chromosome 14 open reading frame 45
1	COPS5	COP9 constitutive photomorphogenic homolog subunit 5
1	CUL1	cullin 1
1	MRPS9	mitochondrial ribosomal protein S9
1	N4BP2L2	NEDD4 binding protein 2-like 2
1	ZNF350	zinc finger protein 350
1	ANKRD7	ankyrin repeat domain 7
1	FMO2	flavin containing monooxygenase 2 (non-functional)
1	PEAK1	NKF3 kinase family member
1	PNISR	PNN-interacting serine/arginine-rich protein
1	SV2B	synaptic vesicle glycoprotein 2B
1	intergenic	chromosome 3 genomic contig
1	ANAPC1	anaphase promoting complex subunit 1
1	ATP2C1	ATPase, Ca++ transporting, type 2C, member 1

bioRxiv preprint doi: https://doi.org/10.1101/2025.05.29.656827; this version posted May 29, 2025. The copyright holder for this preprint (which was not certified by peer review) is the author/funder, who has granted bioRxiv a license to display the preprint in perpetuity. It is made available under aCC-BY 4.0 International license.

1	CD109	CD109 molecule
1	COMMD8	COMM domain containing 8
1	F13A1	coagulation factor XIII, A1 polypeptide
1	OLFML3	olfactomedin-like 3
1	SSR1	signal sequence receptor, alpha
1	UFM1	ubiquitin-fold modifier 1
1	VDAC2	voltage-dependent anion-selective channel protein
1	WWOX	2 WW domain containing oxidoreductase
1	ZBRK1	zinc-finger protein ZBRK1
1	ZNF177	zinc finger protein 177
1	ZNF237	zinc finger protein 237
1	ZNF251	zinc finger protein 251

bioRxiv preprint doi: https://doi.org/10.1101/2025.05.29.656827; this version posted May 29, 2025. The copyright holder for this preprint (which was not certified by peer review) is the author/funder, who has granted bioRxiv a license to display the preprint in perpetuity. It is made available under aCC-BY 4.0 International license.

Supplementary Table 3. Bacterial strains used in this study.

**Bacterial strain**	**Genotype**	**Reference**
Shigella flexneri srv. 5a str. M90T	Naturally Sm^{R}	[8]
S. flexneri mCherry	Constitutively producing mCherry, Carb^{R}, Sm^{R}	[84]
S. flexneri GFP	Constitutively producing GFP, Carb^{R}	[8]
S. flexneri afaI	Constitutively producing the adhesin AfaE, Amp^{R}, Sm^{R}	[8]
S. flexneri pNVgfp afaI	Displaying an anti-GFP nanobody on the bacterial surface upon induction with IPTG, constitutively producing the adhesin AfaE, Amp^{R}, Cm^{R}, Sm^{R}	This study
S. flexneri Δ*mxiE* Ruby	*mxiE*$^{-}$*,* constitutively producing Ruby, Carb^{R}, Sm^{R}	(Gift from Felix Randow lab)
S. flexneri Δ*mxiE pNVgfp afaI*	*mxiE*$^{-}$*,* displaying an anti-GFP nanobody on the bacterial surface upon induction with IPTG, constitutively producing the adhesin AfaE, Amp^{R}, Cm^{R}, Sm^{R}	This study
S. flexneri Δ*rfaC*	*rfaC*$^{-}$, SmR	[38]
S. flexneri Δ*ipaH9.8*	*ipaH9.8*$^{-}$, Kan^{R}, Sm^{R}	This study
S. flexneri Δ*rfaC* Δ*ipaH9.8*	*rfaC*$^{-}$, *ipaH9.8*$^{-}$, Kan^{R}, Sm^{R}	This study
S. flexneri Δ*rfaC* GFP	*rfaC*$^{-}$, constitutively producing GFP, Carb^{R}, Sm^{R}	This study
S. flexneri Δ*rfaC* mCherry	*rfaC*$^{-}$, constitutively producing mCherry, Carb^{R}, Kan^{R}, Sm^{R}	This study
S. flexneri Δ*ipaH9.8* GFP	*ipaH9.8*$^{-}$, constitutively producing GFP, Carb^{R}, Kan^{R}, Sm^{R}	This study
S. flexneri Δ*ipaH9.8* Ruby	*ipaH9.8*$^{-}$, constitutively producing Ruby, Carb^{R}, Kan^{R}, Sm^{R}	(Gift from Felix Randow lab)
S. flexneri Δ*rfaC* Δ*ipaH9.8* GFP	*rfaC*$^{-}$, *ipaH9.8*$^{-}$, constitutively producing GFP, Carb^{R}, Kan^{R}, Sm^{R}	This study
S. flexneri Δ*rfaC* Δ*ipaH9.8* mCherry	*rfaC*$^{-}$, *ipaH9.8*$^{-}$, constitutively producing mCherry, Carb^{R}, Kan^{R}, Sm^{R}	This study
Escherichia coli DH5a	F– φ80lacZΔM15 Δ(lacZYA-argF) U169 *rec*A1 *end*A1 *hsd*R17(r$_{K}^{-}$, m$_{K}^{+}$) *pho*A *sup*E44 λ^{-} *thi*-1 *gyr*A96 *rel*A1	Thermo Fisher Scientific (#18265017)
E. coli BL21 (DE3)	F– *ompT gal dcm lon hsdSB* (*rB*$^{-}$ *mB*$^{-}$) pLysS, Cm^{R}	Thermo Fisher Scientific (#EC0114)
Salmonella enterica subs. *enterica* srv. Typhimurium str. SL1344 pmCherry	For the arabinose inducible expression of mCherry	This study

bioRxiv preprint doi: https://doi.org/10.1101/2025.05.29.656827; this version posted May 29, 2025. The copyright holder for this preprint (which was not certified by peer review) is the author/funder, who has granted bioRxiv a license to display the preprint in perpetuity. It is made available under aCC-BY 4.0 International license.

Supplementary Table 4. Plasmids used in this study

Plasmid	Relevant characteristics	Reference
pKD46	*repA101*(ts) *oriR101* bla *ParaB*l-Red recombinase, Carb^R.	[67]
pKD4	oriR$_{\gamma R6k}$ *FRT::kan::FRT*, Kan^R.	[67]
pCP20	For elimination of resistance gene.	[67]
pFPV25.2	For constitutive expression of GFP.	[85]
pFPV-mCherry	For constitutive expression of mCherry.	[86]
pmCherry	Derivative of pFUS-PBAD[69]. For arabinose inducible expression of mCherry.	This study
pAC-*afaI*	For constitutive expression of the bacterial adhesin AfaE, ori p15A, Amp^R.	[72]
pNVgfp	For IPTG inducible expression of anti-GFP nanobody on *S. flexneri* surface [Intimin$_{EHEC}$ (1-659)-E-Vgfp-myc tag].	[71]
pTRC-GFP-APEX2	Derivative of pTRC-APEX2[37], msGFP fused the N-terminus of APEX2.	This study
pGEX6P-1 GST-IpaH9	For the purification of GST-IpaH9.8. Amp^R	(Gift from Neal Alto lab)
pGEX6P-1 GST-ubiquitin	For the purification of GST-Ubiquitin. Amp^R	(Gift from David Komander lab)
pJB179-10	Derivative of pGKBT7 vector. IpaH9.8 C337A bait vector for Yeast-Two-Hybrid. Neo^R	This study
pRK5Myc-IpaH9.8	Derivative of pRK5 expressing Myc-IpaH9.8 for the immunoprecipitation experiments. Carb^R.	This study
pCR3.1His-UFM1	Derivative of pCR3.1 expressing His-UFM1. for immunoprecipitation experiments. Carb^R	This study
pLenti-X1-Neo-HA-UFL1	Lentiviral expression of HA-tagged human UFL1. Carb^R.	[30]
pRK5-HA-UFM1-dCS	Mammalian expression of HA-tagged human UFM1-cCS Carb^R.	[30]
pDEST-eGFP-RNF213	For mammalian expression of eGFP-RNF213, Kan^R.	[87]

bioRxiv preprint doi: https://doi.org/10.1101/2025.05.29.656827; this version posted May 29, 2025. The copyright holder for this preprint (which was not certified by peer review) is the author/funder, who has granted bioRxiv a license to display the preprint in perpetuity. It is made available under aCC-BY 4.0 International license.

Supplementary Table 5. Primers used in this study.

**Primer**	**Sequence (5'-3')**	**Use**
del-ipaH9.8-fw	gtaatttcctcactgagctaccagcattttctgagggaaatagtgtag gctggagctgcttc	Deletion of *ipaH9.8*
del-ipaH9.8-rv	accaggagggttttccggagattgttccatgtgagcgcgacacatg ggaattagccatggtcc	Deletion of *ipaH9.8*
comp-ipaH9.8-fw	gtaatttcctcactgagctaccagcattttctgagggaaata	Confirmation of *ipaH9.8* deletion
comp-ipaH9.8-rv	accaggagggttttccggagattgttccatgtgagcgcgac	Confirmation of *ipaH9.8* deletion
msGFP-fw	ccaccaccacatgagtaaaggtgaagaac	Amplification/cloning msGFP into pTRC-APEX2
msGFP-rv	aagactttccaaagtagagttcatccatg	Amplification/cloning msGFP into pTRC-APEX2
pTRC-APEX2-fw	actctacaaaggaaagtcttacccaactgtgag	Amplification/cloning of pTRC-APEX2
pTRC-APEX2-rv	ctttactcatgtggtggtggtggtggtg	Amplification/cloning of pTRC-APEX2
scramble target 1	taatacgactcactataggcaggcaaagaatccctgccgttttaga gctagaaatagc	Zebrafish Cas9-mediated KD
scramble target 2	taatacgactcactataggtacagtggacctcggtgtcgttttagag ctagaaatagc	Zebrafish Cas9-mediated KD
scramble target 3	taatacgactcactataggcttcatacaatagacgatggttttagag ctagaaatagc	Zebrafish Cas9-mediated KD
scramble target 4	taatacgactcactataggtcgttttgcagtaggatcggtttagagct agaaatagc	Zebrafish Cas9-mediated KD
ufl1 target_1	taatacgactcactataggagctcatcacgaatttccgttttagagct agaaatagc	Zebrafish Cas9-mediated KD
ufl1 target_2	taatacgactcactatagggctgggcagaataatccagttttagag ctagaaatagc	Zebrafish Cas9-mediated KD
ufl1 target_3	taatacgactcactataggagacgacgcacagctcacgttttagag ctagaaatagc	Zebrafish Cas9-mediated KD
ufl1 target_4	taatacgactcactataggttcatacctcgctggtcagttttagagct agaaatagc	Zebrafish Cas9-mediated KD
ufm1 target_1	taatacgactcactataggacgctgacgtcggacccggttttagag ctagaaatagc	Zebrafish Cas9-mediated KD
ufm1 target_2	taatacgactcactatagggcagaagaggtgagttgagttttagag ctagaaatagc	Zebrafish Cas9-mediated KD
ufm1 target_3	taatacgactcactatagggatggcactggtagctgcgttttagagc tagaaatagc	Zebrafish Cas9-mediated KD
ufm1 target_4	taatacgactcactataggcaatccggcgcagacagcgttttagag ctagaaatagc	Zebrafish Cas9-mediated KD
sgRNA Scaffold Primer	aaaagcaccgactcggtgccactttttcaagttgataacggactag ccttattttaacttgctatttctagctctaaaac	Zebrafish Cas9-mediated KD
ufl1 fw	acgtgcagttcctctcagtg	qRT-PCR
ufl1 rv	acttgtcgacaggaaggctg	qRT-PCR
ufm1 fw	gcggacctctacctttgctt	qRT-PCR
ufm1 rv	acttctcccctttccccagt	qRT-PCR
ef1a1l fw	aagcttgaagacaaccccaagagc	qRT-PCR
ef1a1l rv	actcctttaatcactcccaccgca	qRT-PCR

bioRxiv preprint doi: https://doi.org/10.1101/2025.05.29.656827; this version posted May 29, 2025. The copyright holder for this preprint (which was not certified by peer review) is the author/funder, who has granted bioRxiv a license to display the preprint in perpetuity. It is made available under aCC-BY 4.0 International license.

Extended Data Figures

Extended Data Fig. 1. Functionalisation of *S. flexneri* with GFP-APEX2 and proximity biotinylation of bacterial surface in vitro. a, Diagram representing the GFP-APEX2 construct for *E. coli* expression and subsequent purification. **b,** Purification of GFP-APEX2. Coomassie stained SDS-PAGE showing protein ladder for molecular weight (MW), clarified extract (CE), flowthrough (FT), elution fractions (E) and purified protein (PP). Fractions E2 to E5 were pulled together. Resulting GFP-APEX2 protein has a predicted molecular weight of 55.69 KDa. **c,** Diagram showing the experimental method. First, the expression is anti-GFP nanobody on *S. flexneri* surface is induced with IPTG. Then, bacteria are functionalised in vitro with GFP-APEX2. Finally, the proximity biotinylation reaction happens after addition of BP and H_2O_2. **d,** Representative airyscan confocal images showing biotinylation at the vicinity of functionalised *S. flexneri* in vitro, in the presence or absence of IPTG and H_2O_2. SAV stands for streptavidin. Scale bar, 1 µm. **e,** Quantification of GFP-APEX2 coating and protein biotinylation at the surface of *S. flexneri* in the presence or absence of GFP-APEX2, BP and H_2O_2, measured by flow cytometry. More than 60,000 bacteria from a total of 3 independent experiments were analysed per condition. The results are represented as median ± interquartile range. Kruskal-Wallis test and Dunn's multiple comparisons test.

Extended Data Fig. 2. Functionalised *S. flexneri* infects HeLa cells. a-b, Representative confocal microscopy images of HeLa cells infected with GFP-APEX2 coated *S. flexneri* forming actin tails and entrapped in septin cages, respectively. **c,** In vitro secretion assay of *S. flexneri* WT and Δ*mxiE* displaying surface nanobodies and producing the AfaE adhesin indicate a functional T3SS upon Congo red stimulation. **d,** *S. flexneri* WT and Δ*mxiE* displaying surface nanobodies and producing the AfaE adhesin have similar HeLa invasion rates. The results are represented as mean ± SD.

bioRxiv preprint doi: https://doi.org/10.1101/2025.05.29.656827; this version posted May 29, 2025. The copyright holder for this preprint (which was not certified by peer review) is the author/funder, who has granted bioRxiv a license to display the preprint in perpetuity. It is made available under aCC-BY 4.0 International license.

Extended Data Fig. 3. *S. flexneri* proxisome leads to identification of novel host factors recruited to bacteria. a, Principal component analysis showing different proxisomes are identified during infection of *S. flexneri* WT and Δ*mxiE.* **b,** Volcano plot of proteins identified. **c,** GO terms for biological process enriched in the proxisome of *S. flexneri* during infection. **d,** STRING diagram displaying the E3 ligases and deubiquitylases identified. Edges represent shared physical complex; strength of the edge represents confidence.

Extended Data Fig. 4. UFL1 and UFM1 are recruited to *S flexneri.* Representative confocal images of HeLa cells expressing HA-UFL1 or HA-UFM1-dCs and infected with *S. flexneri afaI.* Arrows show localisation of exogenous proteins to intracellular bacteria. Scale bar, 10 µm.

Extended Data Fig. 5. Deletion of ipaH9.8 in *S. flexneri* and recruitment UFL1 and UFM1 recruitment to *S. flexneri* and *S.* Typhimurium in HeLa cells. a, Deletion of *ipaH9.8* in *S. flexneri* WT and Δ*rfaC*. Diagram indicating the primer annealing regions for the insertion of a Kanamycin resistance cassette, prior (top) and after (bottom) insertion. **b-c**, PCR showing the insertion of the Kanamycin resistance cassette in *S. flexneri* WT and Δ*rfaC.* **d,** Percentage of *S. flexneri* WT (n=1311, n=837), Δ*ipaH9.8* (n=463, n=576), Δ*rfaC* (n=630, n=721), and double Δ*rfaC*Δ*ipaH9.8* (n=553, n=473) mutants colocalising with UFL1 at 3 and 5 hours post infection (h.p.i), respectively. **e,** Percentage of *S. flexneri* WT (n=882, n=566), Δ*ipaH9.8* (n=370, n=531), Δ*rfaC* (n=643, n=851), and double Δ*rfaC*Δ*ipaH9.8* (n=526, n=650) mutants colocalising with UFM1 at 3 and 5 hours post infection, respectively. The results are represented as mean ± SD. Two-way ANOVA and Tukey's multiple comparison test. **f,** Representative deconvolved widefield image showing HeLa cell expressing GFP-RNF213

bioRxiv preprint doi: https://doi.org/10.1101/2025.05.29.656827; this version posted May 29, 2025. The copyright holder for this preprint (which was not certified by peer review) is the author/funder, who has granted bioRxiv a license to display the preprint in perpetuity. It is made available under aCC-BY 4.0 International license.

infected with *S.* Typhimurium and immunostained for UFL1. Green, magenta and white arrows show bacteria recruited with GFP-RNF213 only, UFL1 only, or both, respectively. Scale bar, 5 µm.

Extended Data Fig. 6. IpaH9.8 immunoprecipitation leads to UFM1 species enrichment. HEK293T cells were transfected with plasmids that express His-UFM1 and/or Myc-IpaH9.8 and harvested. Unbound (UB), wash (W1, W2, W3), and eluted (ELU) fractions were analysed via SDS-PAGE followed by Coomassie stain or immunoblot. His-UFM1 was co-purified with Myc-IpaH9.8 using protein anti-myc antibody agarose beads, versus a His-UFM1-only control. UFM1 species were detected with anti-UFM1 antibodies (BostonBiochem).

Extended Data Fig. 7. UFL1 and UFM1 are recruited to intracellular *S. flexneri* in THP-1 macrophages and in zebrafish larvae. a-b, Representative confocal image of THP-1 macrophages infected with *S. flexneri* WT, Δ*rfaC*, and Δ*rfaC*Δ*ipaH9.8*. Arrows show localisation of UFL1 and UFM1, respectively, to intracellular bacteria. Scale bar, 10 µm. **c-d,** Representative confocal image of zebrafish larvae infected at the tail musculature with *S. flexneri* WT, Δ*rfaC*, and Δ*rfaC*Δ*ipaH9.8* at 2 hours post infection and stained against UFL1 or UFM1. Scale bar, 20 µm and 5 µm for the inset. Arrows show localisation of UFL1 or UFM1 to bacteria. Yellow arrows show bacteria that have partially or completely lost their mCherry fluorescence.

Extended Data Fig. 8. Knockdown of *ufm1* in HeLa cells. a, Western blot showing reduced UFM1 levels after depletion by siRNA in HeLa cells, using anti-UFM1 antibodies (Proteintech). **b,** Quantification of UFM1 upon siRNA depletion detected by Western blot. Student's t-test. **c,** Representative microscopy images of HeLa cells infected with S. *flexneri*

bioRxiv preprint doi: https://doi.org/10.1101/2025.05.29.656827; this version posted May 29, 2025. The copyright holder for this preprint (which was not certified by peer review) is the author/funder, who has granted bioRxiv a license to display the preprint in perpetuity. It is made available under aCC-BY 4.0 International license.

Δ*rfaC*Δ*ipaH9.8* immunostained against UFM1, under control conditions or after siRNA depletion, confirming antibody specificity. **d-f,** Invasion of *S. flexneri*, *S. flexneri* Δ*rfaC*Δ*ipaH9.8* and *S.* Typhimurium in HeLa cells. Invasion is calculated as the ratio of CFUs recovered at 1 hour post infection (internalised bacteria) to the CFUs of the inoculum. Student's t-test. **g-i,** Intracellular growth of *S. flexneri*, *S. flexneri* Δ*rfaC*Δ*ipaH9.8* and *S.* Typhimurium in HeLa cells, normalised to 1 hour post infection. The results are represented as mean ± SD.

Extended Data Fig. 9. Characterisation of *ufl1* and *ufm1* CRISPR/Cas9 knockdown zebrafish larvae. a-b, Quantification of *ufm1* and *ufl1* expression in zebrafish larvae upon CRISPR/Cas9 knockdown by qRT-PCR. Values are normalised to the median of the control (scramble) condition. The results are represented as mean ± SD. Student's t-test. **c-d,** Representative microscopy images of zebrafish larvae infected with *S. flexneri* at the tail musculature at 2 hours post infection and immunostained against UFM1 and UFL1, respectively, upon *ufm1* and *ufl1* depletion or control conditions, confirming specificity of antibodies. Scale bar, 20 µm. **e,** Representative images of CRISPR Cas9 generated zebrafish larvae with scramble, *ufl1* or *ufm1* gRNAs at 2-, 3- and 5-days post fertilisation (d.p.f.).

Extended Data Fig. 10. Proposed model for intracellular bacteria UFMylation. UFL1 is recruited to the *S. flexneri* surface, where it mediates the decoration of the bacteria with UFM1 to target them for degradation in a mechanism independent of autophagy. UFL1 recruitment is hampered by bacterial LPS, and the Type 3 secreted effector IpaH9.8 binds to UFM1 to prevent its deposition on *S. flexneri.*

bioRxiv preprint doi: https://doi.org/10.1101/2025.05.29.656827; this version posted May 29, 2025. The copyright holder for this preprint (which was not certified by peer review) is the author/funder, who has granted bioRxiv a license to display the preprint in perpetuity. It is made available under aCC-BY 4.0 International license.

Additional information

Supplementary Video 1. Proximity biotinylation in zebrafish larvae. 3D reconstruction of a confocal microscopy z-stack showing a *Tg(lyzC:DsRed2)*^{nz50} zebrafish larva with fluorescently labelled neutrophils infected at the tail musculature with S. *flexneri* WT functionalised for proximity biotinylation. GFP-APEX2 coated bacteria (green) have been phagocytosed by neutrophils (red). Biotinylated proteins (magenta) appear around phagocytosed bacteria. The 3D grid overlay corresponds to 30 µm intervals.
